# Connectivity and complex systems: learning from a multi-disciplinary perspective

**DOI:** 10.1007/s41109-018-0067-2

**Published:** 2018-06-18

**Authors:** Laura Turnbull, Marc-Thorsten Hütt, Andreas A. Ioannides, Stuart Kininmonth, Ronald Poeppl, Klement Tockner, Louise J. Bracken, Saskia Keesstra, Lichan Liu, Rens Masselink, Anthony J. Parsons

**Affiliations:** 1 Durham University, Durham, UK; 2 Jacobs University, Bremen, Germany; 3 Laboratory for Human Brain Dynamics, Nicosia, Cyprus; 4 Stockholm Resilience Institute, Stockholm, Sweden; 5 The University of South Pacific, Suva, Fiji; 6 University of Vienna, Vienna, Austria; 7 Freie Universität Berlin, Berlin, Germany; 8 Leibniz-Institute of Freshwater Ecology and Inland Fisheries, Berlin, Germany; 9 Austrian Science Funds, Berlin, Germany; 10 WageningenUR, Wageningen, Netherlands; 11 University of Sheffield, Sheffield, UK

**Keywords:** Connectivity Studies, Fundamental Unit, Emergent Behaviour, Structural Connectivity, Functional Connectivity, Measuring Connectivity

## Abstract

In recent years, parallel developments in disparate disciplines have focused on what has come to be termed *connectivity*; a concept used in understanding and describing complex systems. Conceptualisations and operationalisations of connectivity have evolved largely within their disciplinary boundaries, yet similarities in this concept and its application among disciplines are evident. However, any implementation of the concept of connectivity carries with it both ontological and epistemological constraints, which leads us to ask if there is one type or set of approach(es) to connectivity that might be applied to all disciplines. In this review we explore four ontological and epistemological challenges in using connectivity to understand complex systems from the standpoint of widely different disciplines. These are: (i) defining the fundamental unit for the study of connectivity; (ii) separating structural connectivity from functional connectivity; (iii) understanding emergent behaviour; and (iv) measuring connectivity. We draw upon discipline-specific insights from Computational Neuroscience, Ecology, Geomorphology, Neuroscience, Social Network Science and Systems Biology to explore the use of connectivity among these disciplines. We evaluate how a connectivity-based approach has generated new understanding of structural-functional relationships that characterise complex systems and propose a ‘common toolbox’ underpinned by network-based approaches that can advance connectivity studies by overcoming existing constraints.

## Introduction

In recent years, parallel developments in disciplines as disparate as Systems Biology, Neuroscience, Geomorphology, Ecology and Social Network Science have focused on what has come to be termed *connectivity*. In its simplest form, connectivity is a description of the level of connectedness within a system, and corresponds to a structured set of relationships between spatially and/or temporally distinct entities (Kool et al., 2013). In these disciplines connectivity has been a transformative concept in understanding and describing what are considered to be complex systems. Complex systems often exhibit non-linear relations between constantly changing components that together form the behaviour of the whole (emergent behaviour) via dynamical relations across multiple levels of organization and scale (Bar Yam, 1997; Cummings and Collier, 2005; Manson, 2001; Wu, 1999), in contrast with simple, non-complex systems, which tend to exhibit linear relations among components. There has been a wealth of research into complex systems in recent decades, perhaps spurred on by the discovery that apparently chaotic behaviour can be generated from simple ‘rules’ (e.g. May 1976).

Whilst conceptualisations and operationalisations of connectivity have evolved largely within their disciplinary boundaries, similarities in the concept and its application among disciplines are also evident. Existing approaches to the study of connectivity used across different disciplines have been applied in different ways to understand system properties that lead to behaviours characteristics of complex systems. We therefore ask the question: Can the concept of connectivity provide insight into some of those simple rules governing complex systems? We explore how approaches used to study connectivity and system dynamics can be used to understand the ‘simple rules’ governing complex systems, and if a common set of approaches can be usefully applied to all disciplines.

To address this question we drew together experts from Systems Biology, Neuroscience, Computation Neuroscience, Geomorphology, Ecology and Social Network Science in a face-to-face-workshop to discuss relationships between these disciplines and connectivity studies, and how we can go about using and sharing connectivity tools among disciplines. In this workshop we identified four common ontological and epistemological challenges in using connectivity to understand complex systems. These challenges form the subject of this review (Table 1).

**Table 1:** Summary of connectivity challenges across different disciplines. Extent to which connectivity challenges are an issue: * do not present a challenge; ** presents a challenge but progress has been made; *** presents a major challenge.

	Fundamental Unit (FU)	Separating Structural Connectivity (SC) and Functional Connectivity (FC)	Understanding Emergent Behaviour	Measuring Connectivity
**Systems Biology**	** • FUs are biologically well defined, based on the biological system rather than measurement process. • A full inventory of functional elements is still missing; also interdependences between different levels of cellular organizations (i.e. among fundamental units from different networks) are often neglected.	** • Generally, a clear time scale separation ensures a distinction between SC and FC. • While FC is a network representation of cellular states (e.g. correlations between metabolite concentrations or gene expression levels), SC is shaped by evolution on a much slower time scale.	** • Concepts such as modularity and hierarchy may provide a starting point for addressing emergent behaviour within the current knowledge of SC and FC, but a true incorporation of the many (spatial and temporal) scales will require novel multiscale methods. • Key challenge: understanding the relation of regulatory mechanisms and emergent collective behaviour.	** • Explosive growth of high-throughput methods providing access to many facets of structural and functional connectivities, but has led to a dramatic diversification of databases, methods and nomenclatures, resulting in a strong need of data and method integration.
**Neuroscience**	* • Defining the fundamental unit of the brain is clear-cut and depends on the level (scale) at which one is working. • The fundamental unit is commonly defined as being the cortical area, although is alternatively defined as the neuron.	** • Different techniques are used to distinguish between SC and FC, but FC is defined (using various techniques) using indirect correlates of brain activity. • Memory affects the relation between SC and FC through its long-lasting anatomical import on the structure of the network. • Understanding the effects of memory remains a key challenge because of the diffuse nature of the anatomical imprint.	** • Great progress has been made in understanding concepts such as attention and emotion as emergent properties of neural activity. • The big challenge today is to push this understanding to its limit and offer a convincing account of how neural activity and its organization in the networks of the brain eventually leads to consciousness.	** • A range of techniques exist to measure connectivity • Due to differences in spatial and temporal resolution between different measurement techniques, hybrid approaches are becoming popular to overcome the challenge of measuring SC and FC at appropriate resolutions. • The main challenge with the [premature] use of hybrid methods is that the final result may be limited to what the least sensitive method can provide. • Measurements of FC are inferred from high-resolution snapshots, rather than being measured directly.
**Computational Neuroscience**	* • The fundamental unit is typically defined as being individual neurons or cortical areas and form the nodes of a network. Identifying these fundamental units is done using anatomical means and neurobiological knowledge.	** • It is increasingly common to directly and quantitatively compare SC and FC (such separations are readily undertaken and pose no major challenges). • A limitation is that SC/FC correlations only tend to look in one direction only – the effect of structure on function.	* • Network-based approaches are used, allowing for the interplay of SC and FC allow for the exploration of self-organization and pattern formation – both important characteristics of emergent behaviour.	* • Measurements of connectivity are undertaken using a wide range of network-based descriptors of connectivity.
**Geomorphology**	*** • No clear or consistent definition of the fundamental unit (it is dependent on the research question).	*** • Separating SC and FC in a meaningful way is challenging because of the myriad of processes operating over a multitude of spatial and temporal scales. • Separating SC and FC is compounded by the imprint of memory and timescales over which it affects connectivity for a meaningful separation of SC and FC.	*** • Tools to explore how SC and FC lead to emergent behaviour are lacking. • Multi-method approaches focussing on the interactions of SC and FC over relevant spatio-temporal scales may aide in understanding emergent behaviour.	** • Approaches to measure SC are well developed, and make use of high-resolution techniques where appropriate. • No direct measurement techniques for FC are available and rely on inferring connectivity from snapshots of information. • Modelling is often used as a surrogate for direct measurements of FC.
**Ecology**	** • Conceptually the fundamental unit is defined as the ecosystem. • In operational terms, defining the FU is more difficult.	** • Linking and separating SC and FC is common, but made challenging where there are time lags in the response of ecological function to changes in ecological structure and vice versa.	** • Many attempts to explain emergent behavior (e.g. using advection-diffusion models) have produced realistic patterns, but at the expense of realistic processes. • A challenge is to study emergent behaviour using model structures that are not inherently designed to produce patterns.	** • A range of techniques exist for measuring SC based on simple indices of patch connectivity, through to network-based approaches. • Measuring FC poses much more of a challenge and requires dealing with complex phenomena that are difficult to quantify and tend to reply upon inferring FC connectivity based on a series of empirical measurements through time. • Advances being made in measuring SC and FC through the use of weighted monopartite and bipartite networks.
**Social Network Science**	* • Traditionally the fundamental unit is defined as the person, although more recently the definition has become less certain with some researchers now using the interaction as the unit of study.	*** • The focus tends to be on the connectivity of structural networks. • Approaches to look at dynamics (FC) are limited in terms of analytical power. • In social networks culture is a type of memory effect that affects function (i.e. the response of an individual to social interactions), and the complexity that an evolving mix of cultures brings to social networks is a significant challenge.	*** • The emergence of the network property is conditional on entire network interactions, and the challenge of adopting models of social behaviour that recognise the diversity of social interactions across a population complicates matters further.	*** • Measuring connectivity is a major challenge due to ethical, practical and philosophical constraints.

## Four key challenges in using connectivity to understand complex systems

### Defining the Fundamental Unit

A fundamental unit can be either a physical object or a concept that sustains its identity and participates in the interactions within a system for a sufficiently long time and in sufficiently important ways to merit quantification. Explicit definition of the fundamental unit is required when studying connectivity. In cases where the fundamental unit is defined based on a concept, a way of operationalising that concept is required. *A key challenge is defining the appropriate fundamental unit for a specific application*, and often depends on the [natural] scales at which it is conceptually robust to work for a specific application. Hierarchical organization is a common feature of complex systems, and typically this refers to a nested, module-within-module structure. Each hierarchical level may, in principle, enable us to make meaningful choices about the fundamental unit for a given application. Each level of such a hierarchical structure could be used to define the fundamental unit. For example, in neuroscience the fundamental unit could be an individual neuron, or a cortical area. However, within some disciplines the scalar boundaries are often fuzzy and identification of clear hierarchical structures is difficult.

### Separating Structural Connectivity and Functional Connectivity

Approaches to the study of connectivity within complex systems have often addressed structure (network architecture) and function (dynamical processes) separately. Structural connectivity (SC) measures of a system are used to quantify the level of configuration or arrangement of a network, whilst the functional connectivity (FC) of a system describes dynamical processes operating within a structurally connected network. SC thus derives from the system’s anatomy, whereas FC is inferred from the system’s process dynamics which are represented by fluxes and transformations of energy, matter or information between structural units. Structure always affects function (Strogatz, 2001), and often (but not always) function affects structure, although the timescales of the reciprocity/feedback may differ. Many connectivity-based approaches separate SC and FC in order to simplify their study. However, this separation is challenging because the degree of connectivity and feedbacks between SC and FC will depend on the spatial and/or temporal scale(s) at which the system is studied: as spatial scale increases connectivity becomes an internal process that cannot be represented or quantified explicitly. For example, in Ecology connectivity amongst different patches cannot be explicitly represented when studying the system at a higher level or organization. In many (if not most) systems, SC evolves over time. For example, in Neuroscience, although the structure of the child’s brain appears similar to that of the adult brain, its size, orientation within the cranium and details of the anatomical connectivity changes appreciably over the next few years and some areas of the brain, especially in the frontal lobe, only mature after adolescence. Capturing this evolution is a fundamental advance provided by complexity-based approaches compared to more traditional systems-based concepts. At different timescales the relation between SC and FC may look very different. Thus, a key issue when separating SC and FC is determining the timescale at which a change in SC becomes dynamic (i.e., functional), and this in turn may depend on the fundamental unit. System memory – the imprint that function leaves on the structure of a system or network – also affects the interplay of SC and FC, and therefore a further consideration is how to incorporate memory into quantitative descriptions of the structural-functional evolution of connectivity. The separation of SC from FC is artificial and is thus subjective. *A key challenge in separating SC and FC is weighing up the gains that can be made by making these separations, versus the losses in our potential understanding of the system that arise due to these separations.* These gains and losses depend on the timescale of interest compared to the rate of evolution of system structure.

### Understanding emergent behaviour

The structural-functional evolution of a system via structural-functional connectivity may lead to emergent behaviour whereby local interactions lead to self-organised phenomena observable at larger spatial scales that cannot be predicted (or at least they are not obvious at the local level: what Bedau (1997) calls “weak emergence”). Emergent behaviour is an important characteristic of complex systems that leads to the formation of patterns; simple and complex patterns at the larger scale can be formed by very simple interactions at the local level. These simple interactions depend on the local connectivity of entities within a system. However, deriving quantitative descriptions of emergent behaviour is challenging. Theoretical tools to relate emergent behaviour to structure and function are lacking, and by separating SC from FC we make it even more difficult to analyse emergent behaviour. *A key challenge is how to best use concepts and operalizations of connectivity to develop quantitative descriptions of emergence that will enable us to understand emergent behaviour better, and overcome the aforementioned barriers to this goal.*

### Measuring connectivity

To apply the concept of connectivity to understand the behaviour of real-world complex systems, it is necessary to quantify connectivity, yet this poses several challenges. The level of connectivity observed within a system depends on the lens through which one observes it. Due to the inability of existing tools to measure connectivity directly, it is often necessary to infer connectivity from an alternative set of indirect measurements. Indirect measurements can lead to subjectivity and uncertainty in our understanding of connectivity and dynamical feedbacks operating within a system, and furthermore, can impede appropriate operational definitions of the fundamental unit. There is also a timescale issue: at finer timescales measurements of connectivity may tend towards descriptors of SC rather than FC. FC is more than just inferring what is happening between snap-shots, but trying to determine the actual processes operating to produce fluxes (Bracken et al., 2013). How many snapshots do we need of a system, and how close in time do they need to be before we can be confident to capture the “dynamic or functional” aspect of connectivity? A variety of tools for studying connectivity exist: *the challenge here is both to recognise the limitations that these tools place on our understanding of connectivity, and to develop new tools where those limitations significantly constrain our understanding.*

## Disciplinary perspectives

Within all the disciplines explored here, an abstraction of the system is required in order to study connectivity. Most commonly, this abstraction involves representing the system as a network. In recent years networks have emerged as an invaluable tool for describing and quantifying complex systems, especially due to the ease with which networks can be used to represent hierarchical organization over multiple scales (Clauset et al., 2008). The representation of a system as a network provides the opportunity – using network-based tools – to disentangle noise and stochasticity from non-random patterns and mechanisms, in order to gain a better understanding of how these systems function and evolve (Menichetti et al., 2014). Here, the network-based approach underpins our conceptual framework for connectivity studies due to its ubiquity.

The simplest network-based abstraction of a system is a graph consisting of nodes and links. More detailed abstractions can be achieved through the use of weighted links and directional links. In an unweighted network, links are binary entities, where they are either present or not. In a weighted network links have associated weights that record their strengths relative to one another (Newman, 2004a). In a directional network, the flow of information or material is in one direction, from one node to another, whereas in an undirected network, flow is in either direction between two nodes. A system can also be represented as a bipartite network, in which two different classes of nodes can be represented, and links between nodes are allowed across but not within two classes (Daminelli et al., 2015), thus allowing identification of interactions between distinct groups of nodes. Furthermore, whilst some disciplines can be represented using non-spatial networks, space is critical in others.

**Fig. 1:**
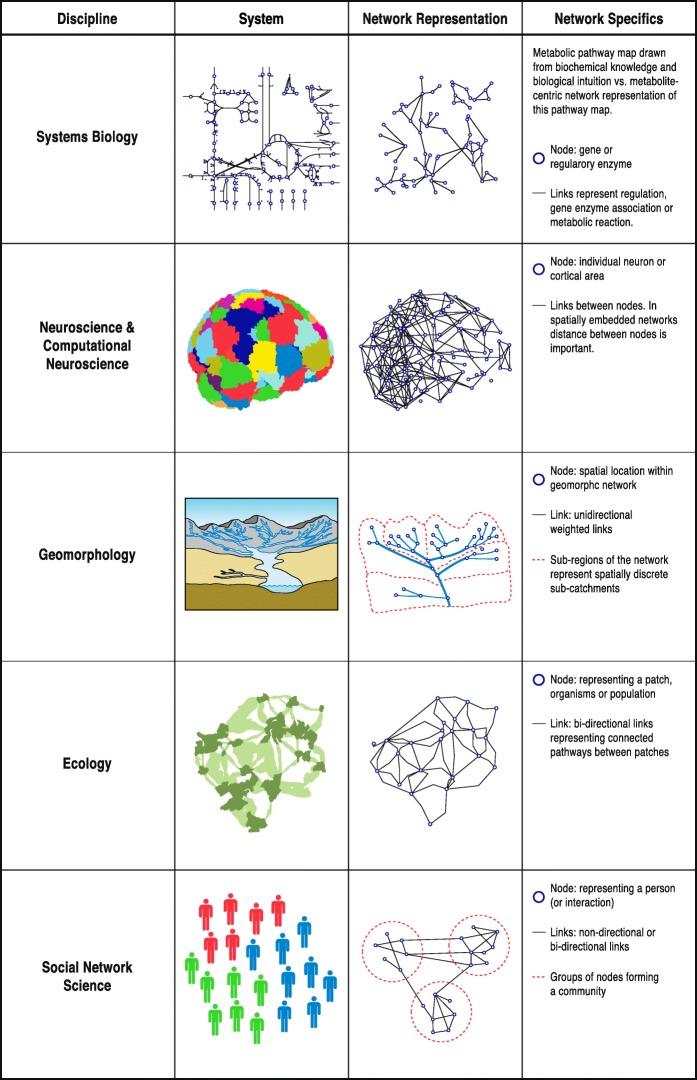
Network-based representation of structural and functional connectivity. Illustration of ways in which structural and functional connectivity within a multitude of systems can be conceptualised using a network-based approach across Systems Biology, Neuroscience/Computational Neuroscience, Geomorphology, Ecology, and Social Network Science

A wide range of different types of systems can be represented as a network (Fig. 1), which raises the possibility for a set of common tools and opportunity for the more general, trans-disciplinary study of SC and FC using network-based approaches.

### Systems Biology

Systems Biology strives to understand biological entities as complex systems through analysis of the interdependencies of the components of these systems; i.e., via a system’s *connectivity*. Over the past two decades, new high-throughput technologies have enabled mathematical modelling, statistical analysis and numerical simulation of biological systems with unprecedented detail for each level of description. At the core of systems thinking in Biology is the concept of networks (see, e.g., Barabasi and Oltvai, 2004; Barabasi et al., 2011; Cowen et al., 2017; Hütt, 2014). Together with Statistical Physics (e.g., Albert and Barabasi, 2002), Systems Biology has been one of the principal drivers behind the development of a theory of complex networks and a rich set of methods for network analysis (Barabasi and Oltvai, 2004).

#### Defining the Fundamental Unit

An important prerequisite for the success of networks in Systems Biology is that the fundamental units are biologically well defined. Metabolic networks, for example, are a compilation of biochemical knowledge (the set of chemical reactions catalyzed by enzymes) and the inventory of genes encoding enzymes derived from a carefully annotated genome. The most prominent category of gene regulatory networks, transcriptional regulatory networks, is obtained from genes in the genomes together with the binding sites for transcription factors. As is often the case, many little issues are hidden in the details underneath these general definitions. For example, it is well known that a distinction between main metabolites and ’currency metabolites’, which balance proteins, energy and other chemical factors, is decisive, when evaluating the statistical properties of metabolic networks (Ma and Zeng, 2003). Similarly regarding gene regulatory networks, there is a multitude of other regulatory mechanisms in addition to transcription factors. Thus, even in the comparatively simple case of bacterial gene regulation, we cannot assume the transcriptional regulatory network to be a complete representation of the regulatory apparatus (see for example the debate in Marr et al., 2008; Sobetzko et al., 2012). Even though the networks compiled at the gene-regulatory, metabolic or protein interaction level can be thought of as vast compilations of biological information, it should be noted that the underlying databases will drift with time: more biological knowledge is accumulated, but also the biological categories used to derive suitable network representations of the system will be refined and altered. As a consequence, the network properties will drift as well (Beber et al., 2016). Additionally, the diversity of databases, data formats, models and computational tools and the lack of standards on these levels is currently an enormous barrier to progress in the field (Chavan et al., 2011). Thus, when adopting a network representation many details are necessarily omitted, and decisions about these details can have a severe effect on network structures.

#### Separating SC and FC

With the availability of high-throughput (often called ‘omics’) data, attention in the life sciences has shifted from individual components (single molecules, proteins or genes) to system-level descriptions. The challenge for Systems Biology is to derive and interpret detailed ‘multi-omics’ representations of biological systems (Bauer et al., 2016). A key strategy in addressing this challenge is to separate the interdependencies of system components (i.e. SC) from correlations among dynamical observations such as gene activities or metabolite concentrations (i.e. FC). For a wide range of organisms in all domains of life, the genome (i.e., the DNA sequence of one or several representatives of a species) is now available. Typical examples of omics data are transcriptome profiles (the simultaneous measurements of the expression levels of many genes in a cell), proteome profiles (the simultaneous measurements of many/all proteins in a cell), metabolomics profile (the measurement of many metabolite concentrations in a cell) or epigenetic patterns (a genome-wide assessment of methylations and/or histone modifications, which are the most common forms of epigenetic signals).

The richness of biology in terms of molecular components and interactions has for a long time prevented the development of systems-level descriptions. For a long time the detailed mathematical modelling of small subsystems (e.g., metabolic pathways or the interaction of a small number of genes) has been the method of choice for the step from components to (small) systems. This is the defining feature of Theoretical Biology. The abstraction of biological systems in terms of nodes and links has paved the way for more qualitative approaches, which, however, allow us to address the scale of a whole cell. These network approaches have become one of the cornerstones of Systems Biology.

One of the reasons for the success of the network view in Systems Biology is that structural properties and snapshots of biological function are typically measured in independent ways: transcriptome profiles as activity states of gene regulatory networks (which are compiled from information on transcription factors and binding sites), metabolic fluxes or metabolite concentrations as activity states of metabolic networks (which are compiled from known biochemical reactions and the enzymes identified in the genome). For a particular observation of biological activity – for example a transcriptome profile – the effective networks, representing the currently active part of a biological system, can be viewed as a representation of FC.

Comparisons of SC and FC provide relationships either between network representations of global biological knowledge and high throughput ’omics’ data or between different categories of ’omics’ data, each represented by a network. A rich set of relationships between SC and FC has been established in Systems Biology. Examples include the predictive power of elementary flux modes in metabolism (Stelling et al., 2002), the relationship between hierarchy level and gene essentiality in gene regulatory networks (Yu and Gerstein, 2006), the relationship between node degree and essentiality in protein interaction networks (Jeong et al., 2001), the power law distribution of metabolic fluxes (Almaas et al., 2004, see also above), the network interpretation of gene-enzyme scaling relationships (Maslov et al., 2009), the importance of a spatial embedding of regulatory networks in bacterial gene regulation (Hacker et al., 2017; Marr et al., 2008), the interplay of gene regulation and metabolism as an example of interdependent networks (Klosik et al., 2017; Sonnenschein et al., 2011) and the predictability of likely environments from metabolic networks (Borenstein et al., 2008).

As mentioned above, the independence of data resources behind SC and FC are of high relevance to the success of network approaches in Systems Biology. Nevertheless, the possibility of predicting a link (i.e., an element contributing to SC) from (dynamical) data (and hence from information associated with FC) has also been pursued in diverse ways in Systems Biology. Examples of such link prediction or network inference approaches include the inference of gene regulatory networks from gene expression patterns (Marbach et al., 2012) and the inference of microbial interaction networks from species abundances, particularly in the context of (human) microbiomes (Faust and Raes, 2012; Claussen et al., 2017). In the literature on network inference, e.g., in the context of gene expression, methods fall into the following categories (e.g., Le Novere 2015): correlation analysis, methods from information theory, Bayesian inference, and explicit modelling (e.g., using differential equations). In Claussen et al. (2017) an example of an information-theoretical approach is described, where interactions are defined via the entropy shift, when combining binarized abundance vectors using Boolean operations. One should also emphasise that all these methods have been designed for data-rich situations and do not necessarily yield convincing results (see, e.g., the detailed comparison in Marbach et al. (2012) for the case of gene regulatory networks).

In Systems Biology, memory effects in the classical sense of learning and adaptive networks are highly reduced, because the relevant time scales of dynamical behaviour and network adaptation (i.e., cellular function and evolution) are clearly separated. Still, we can expect the networks to be shaped by evolution to optimize or enhance certain functional properties. The precise ’objective function’, however, is not known in detail. In a broader sense, memory effects can be viewed as the presence of slow and fast time scales in a system’s dynamics. Network architecture can facilitate the spread of time scales contributing to a certain biological function, which is seen on the scale of few nodes (e.g., interlinked feedback loops, (Brandman et al., 2005; Brandman and Meyer, 2008)), as well as in the ubiquity of hierarchical and modular network architectures on the metabolic and gene regulatory level (see, e.g., Ravasz et al., 2002; Guimera and Amaral, 2005; Yu and Gerstein, 2006). In Kashtan and Alon (2005) the relationship between modularity and time scales is investigated in more detail.

#### Understanding emergent behaviour

Emergent behaviour is a key concept from the theory of complex systems. In his defining paper on Computational Systems Biology, Hiroaki Kitano emphasized that:

“A popular notion of complex systems is of very large numbers of simple and identical elements interacting to produce ’complex’ behaviours. [In Biology] large numbers of functionally diverse, and frequently multifunctional, sets of elements interact selectively and nonlinearly to produce coherent rather than complex behaviours” (Kitano, 2002).

With this remark, Kitano emphasizes the danger of building up Systems Biology directly from the toolbox of complex systems (see also the more general remark by Keller, 2007). Nevertheless, many examples of self-organised patterns – and thus of emergent behaviour – come from biology, both on the intracellular level (e.g., calcium waves (Falcke, 2004) or the interplay of Min proteins in the *E. coli* cell division (Loose et al., 2008)) and on the multicellular level (e.g., spiral waves and aggregation streams in *Dictyostelium discoideum* (Kessler and Levine, 1993; Palsson et al., 1997)). Networks can play an important role on this level as well. A prominent example is the food foraging network formed by the slime mould *Physarum polycephalum*, which connects spatially distributed food sources in an efficient network layout (Tero et al., 2010). This example points to an important difference between patterns in Physics and Chemistry on the one hand and patterns in Biology on the other. In Physics and Chemistry, patterns are often a by-product of the nonlinear interactions of system components. In Biology patterns often have undergone a clear evolutionary tuning and this might serve a system-level function (such as the network connecting food sources in the case of *Physarum*, the aggregation of cells as a step to a multicellular organism in the case of *Dictyostelium* or the spatial organization of cell division in *E. coli*). The regulatory components must therefore have evolved to yield stable functional patterns and we can thus expect a deep relationship between regulatory components and properties of spatiotemporal patterns. An example is strong and non-monotonous dependence of the density of spiral waves (which regulates the size of the later-stage multicellular aggregates) on the intracellular feedback loop regulating the production of the main signalling substance, cAMP (Sawai et al., 2005). Understanding the relationship of regulatory mechanisms and emergent, collective behaviours is an important future challenge in Systems Biology (Grace and Hütt, 2015).

#### Measuring connectivity

Generally speaking, in Systems Biology the (structural) networks come from two main sources: (1) they are obtained from repositories of accumulated biological information; (2) they are derived from high-throughput data. In the first case, the networks are defined via ‘knowledge accumulation’, rather than by ‘measuring’ connectivity.

As in most other disciplines discussed here, it is highly instructive to discriminate between the cases, where the system properties define the units (i.e., the nodes and links in the network representations of the systems), and the cases, where the measurement technique defines these units. Typically, for networks extracted from accumulated biological information the fundamental units are *not* dictated by the measurement process, but rather by the biological system itself. As outlined above, metabolic networks can be seen as an example of this category. Protein interaction networks, on the other hand, are an example of the other type of SC: There, a link (the physical binding of two proteins) has originally become a fundamental unit due to its accessibility via high-throughput measurements. The biological relevance of such protein-protein interactions is not the defining criterion. In fact, a link between two proteins might be part of the network, even though the two proteins are located in different cellular compartments and therefore will never actually have a chance of interacting.

Functional connectivity, which is a representation of the current state of, for example, a biological cell, is typically measured via the high-throughput technologies discussed above. Such as state can be an activity pattern of all genes or a list of concentrations of metabolites available in the cell at a certain moment in time. Correlations between states and ‘effective networks’ (subnetworks derived from structural networks by only considering active/high-concentration components and their connections) are a typical method for deriving FC from high-throughput data.

### Neuroscience

Neuroscience is the study of the nervous system and is centred on the brain. Connectivity in neuroscience is mainly studied using networks, with a range of networks being studied, depending on what is adopted as the network components. The techniques used by neuroscientists range from molecular and cellular studies of individual nerve cells to imaging of sensory and motor tasks in the brain. These techniques have enabled researchers to investigate the nervous system more fully, including how it is structured, how it works, how it develops, how it malfunctions, and how it can be changed. For the sake of brevity, here we focus on an intermediate level description where the nodes of the network are well circumscribed areas that are bounded by borders that are sharply defined by changes in both structure (anatomically definable changes in the local architecture) and function (e.g. the way some property in the mapping of the external visual field varies smoothly within it and sharply changes as the boundary is crossed).

#### Defining the Fundamental Unit

Within Neuroscience, it is usual to focus on a level of description that is characteristic of the structural organization of the brain and the functions it performs, and for didactic purposes and for practical reasons, it is usual to focus on a network description that has neither too few nor too many elements. There are roughly 100 billion neurons in a brain, so a network description based on individual neurons or sub-cellular entities like synapses will lead to a network of too many components (to be of practical use). When one studies quantitatively the way the constituent neurons are arranged in space with respect to each other (the cytoarchitecture) and what neurotransmitters they express (receptor density distribution) a clear pattern emerges: the brain is divided into a few hundred areas (about 200 to 300 areas for the cortical mantle)^1^. Within each one of these areas the cytoarchitecture and receptor density distributions are fairly uniform with only relatively slow and gradual variation. In contrast, at the borders between these areas there is a rapid transition, so identifying either the rapid changes in cytoarchitecture or receptor density properties allows an accurate and objective identification of the borders and hence an accurate delineation of these areas (Zilles et al., 2002), simply denoted as cytoarchitectonic areas of the brain. For the purposes of the following discussion, the cytoarchitectonic areas are the fundamental unit for neuroscience, at least at the level of nodes for the networks focused on here.

#### Separating SC and FC

The investigation of structural and functional networks of the brain is at the heart of many initiatives in neuroscience; it is an essential component of the Human Brain Project (https://www.humanbrainproject.eu/en/) and it is the primary goal of the Human Connectome Project (http://www.humanconnectomeproject.org/). Functional connectivity *per se* is defined in terms of quantitative measures of linked activity, computed from time series of regional brain activations. Indirect correlates of brain activity are mediated by metabolic changes as these can be traced from the regional consumption of radioactively labelled glucose in Positron Emission Tomography (PET) or the Blood Oxygenated level Dependent (BOLD) functional Magnetic Resonance Imaging (fMRI). Both methods provide indirect correlates of brain activity with time constants of many minutes for PET and a few seconds for fMRI. These changes are slow – orders of magnitude slower than the few millisecond transit time for the activity between areas. With improvements in the accuracy of these methods, it has become clearer that the foci of brain activity coincides with the cytoarchitectonic areas, with initial demonstrations emphasizing responses to well defined stimuli as these excite the early cytoarchitectonic areas in each sensory hierarchy.

As used in the study of complex systems, the term ‘memory’ derives from Neuroscience. Within Neuroscience, memory comes under different forms, each characterised by a different temporal scale. Modality specific (sensory) memory allows continuity in perception and it typically decays within a second. Short-term memory allows us, possibly through rehearsal, to remember recent events and has a characteristic decay time around a minute. Short-term memory is very likely based on FC reverberations of patterns of activity that maintain resemblance to the original pattern for only a short time before they become indistinguishable from the background neural activity leaving no permanent trace. In general, references to memory that are applicable to connectivity are about long-term memory, which allows us to recall events over longer time periods. The process of establishing long term memories is facilitated by the transfer of memory related activity from a temporal store centred around the hippocampus to a more permanent storage in the cortex (Bontempi et al., 1999) with active consolidation going on during sleep (Stickgold, 2005). The consolidated memory must be associated with a change in SC of the network, but its long-lasting anatomical imprint is diffusely stamped in many nodes of the anatomical network. These diffused changes are however organised so that spatiotemporal patterns of electrical activity in the network (re-)construct the experience as and when needed in normal life or as a persistent and inescapable replay of dramatic events in pathology. Understanding memory is a challenge for neuroscience because of the diffuse nature of its anatomical imprint and the labile nature of the electrical activity associated with its recollection; currently, it is difficult to capture in its totality by any one or a combination of the different neuroimaging modalities.

#### Understanding emergent behaviour

Detailed analysis of connectivity patterns of the network can be used to identify sub-networks that correspond to individual sensory and other networks. These sub-networks can be derived from the anatomical structure and the SC (e.g. derived from cytoarchitectonic areas, cortical thickness, receptor density for the delineation of areas and anatomical tracing or white matter density quantification from diffusion tensor imaging for the SC). Large-scale organization also emerges from a decomposition of the full network derived from the FC of resting state fMRI (van den Heuvel et al., 2010), electroencephalography (EEG) (Boersma et al., 2011) and magnetoencephalography (MEG) (de Pascquale et al., 2010) data. Analyses of these data reveal a natural decomposition of the full network into distinct sub-networks. The role of each sub-network is evident from the known specialization of its component nodes. The decomposition based on the resting activity reveals sub-networks for each sensory modality; networks that are known to be critical for the implementation of supramodal cognitive functions including attention, working memory and the default mode network (a network that becomes more active when the subject is not occupied with a specific task or monitoring the external environment). It therefore appears that properties of the mind are correlated with emergent behaviour of the functional networks, and are consistent with the properties of the physical brain, as these were determined by wet brain anatomy and electrophysiology.

At this point in time neuroscience has achieved a fair understanding of how elements necessary for complex purposeful behaviour such as attention and memory are implemented, yet it remains a mystery how these are combined to give rise to consciousness which can be considered as the most significant emergent property of all. Understanding how consciousness emerges, from the activity of neurons and the organization and function of the networks they form, constitutes the Holy Grail of modern neuroscience and perhaps of science of the 21^st^ century. A recent synthesis of results from many neuroimaging studies provides a tentative step in this direction within a unified framework that explains how memory and attention are managed in awake state and sleep states and how they help maintain what appears to be a neural representation of self (Ioannides 2018).

#### Measuring connectivity

Anatomical connectivity relates to the number of connections linking two anatomical nodes, i.e. the whole or specific parts of two cytoarchitectonic areas. Quantifying FC requires methods that can separate the contributions from individual cytoarchitectonic areas at temporal resolutions that are typical of the processing time within and the transfer between areas. The distance between neighbouring cytoarchitectonic areas is typically a few millimetres and the typical transfer time between two areas is of the order of 10 milliseconds.

Indirect approaches to measuring FC between cytoarchitectonic areas involve using classical “wet brain” fibre tracing methods (Zeki 1993) or the more recent non-invasive in-vivo white matter tracing using diffusion tensor imaging. The resulting networks are purely structural, and when they are analyzed, e.g. using clustering based on properties of the nodes (e.g. cortical thickness, neurotransmitter density) and/or links (e.g. white matter density and patterns), functional properties of the underlying networks can be deduced, including predictions about the FC patterns (Honey et al., 2009).

Direct measures of brain activity rely on electrophysiology. The EEG records the electrical potential (difference) on the scalp and the MEG records the magnetic field just outside the head. They are both generated by electrical activity in a very large number of neurons that are activated synchronously and they are arranged in a similar way in space, so that the resulting effect summates constructively. The functional networks of the brain can be constructed from the regional brain activations extracted from the EEG and especially MEG data using similar mathematical processes as the ones used for PET and fMRI. While in principle the EEG and MEG records carry similar information, the analysis of MEG signals requires less detailed modelling of the conductivity profile of the head to identify the generators accurately compared with the EEG analysis. The main difference between the networks derived from metabolic and electrophysiological measures is in the timing: minutes or many seconds for PET and fMRI and milliseconds for MEG and EEG.

**Fig. 2:**
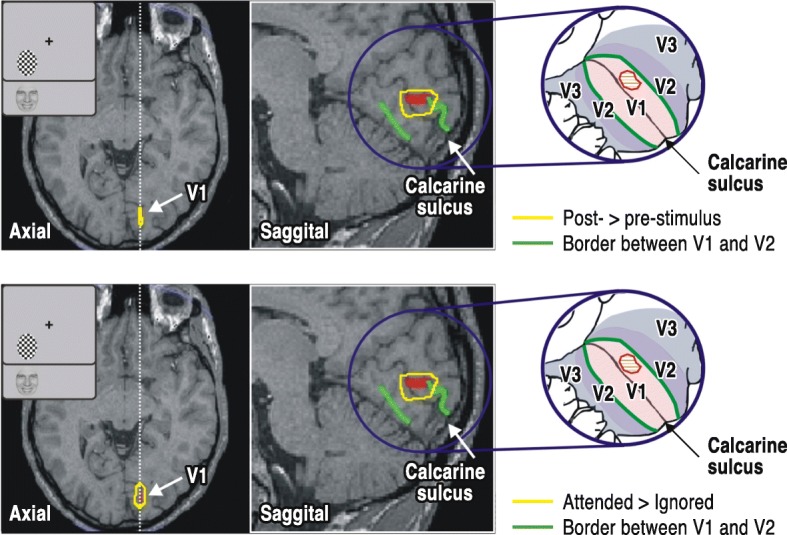
Coincidence in spatial location and timing of activations in the brain. The demonstration of the coincidence in spatial location and timing of the earliest visually evoked (top) and spatial attention (bottom) related activations (responses to images presented in the left visual field). The green lines here indicate the V1/V2 borders (representation of vertical meridian) with the schematic views on the right showing how the activations look on a flat representation of the local cortex, showing clearly the early visual cortex and their boundaries

It is widely believed that techniques relying on slow blood flow have the required spatial resolution but fail on the temporal resolution, while MEG and EEG follow changes in brain activity fast enough but do not have the required spatial resolution. Hybrid approaches are becoming popular in an attempt to bring together the higher temporal resolution of electrophysiology with the perceived more robust localization ability of hemodynamic methods (Babiloni et al., 2004). These hybrid methods rely on simultaneous recording of fMRI and EEG and use EEG to identify periods of specific types of activity and use this to label the corresponding fMRI periods. However, premature integration across different methods and modalities can not only fail to add new information to what the best methods can offer, but can very easily destroy information by limiting the final result to what the least sensitive method can provide. The final description may miss detail (or even fundamental aspects of the organization) that may be available when one or few modalities are pushed to their limits. Magnetic field tomography (Ioannides et al., 1990; Taylor et al., 1999) is a computationally intensive method for extracting accurate estimates of brain activity from MEG data that has been consistently used to push MEG to finer temporal and spatial resolution (Moradi et al., 2003; Poghosyan and Ioannides 2007) so that regional activity (Ioannides 2006) can be studied in great detail and with spatial resolution that allows activity from different cytoarchitectonic areas to be separated. The capability of delineating contributions within distinct cytoarchitectonic areas leads to more refined analysis as in the example shown in Fig. 2 from the first demonstration that spatial attention first influences cortical processing with the first entry of the stimulus induced activity in the primary visual cortex (Poghosyan and Ioannides 2008).

No matter how regional time series are derived, mathematical methods must then be employed to extract from each pair of time series a quantitative measure of the functional link between two brain areas, usually in two stages. First, one must define an appropriate measure of linked activity. For example using time-delayed mutual information as a non-linear measure enables identification and quantification of linkages between areas in real time (for example in relation to an external stimulus or event) and enables assessment of reactive delays. The second stage of addressing the connectivity problem is the technical problem of using graph theory tools to put together the pair-wise links into a more global network. Specific problems can be tackled using a subset of the entire network through judicious choice of what cytoarchitectonic area to include and careful design of experiments.

### Computational Neuroscience

While empirical Neuroscience (section Neuroscience) deals with measuring and functionally interpreting connectivity on many scales, the aspects of Computational Neuroscience, which we address here, deal with structure-function relationships on a more abstract, aggregated level. Generic models of network topology, as well as simple abstract models of the dynamical units, play an important role. In Computational Neuroscience the idea of relating network architecture with dynamics and, consequently, function has long been explored (e.g., Bullmore and Sporns 2009, 2012; Damicelli et al., 2017; Deco et al., 2011). On the level of network architecture, a particularly fruitful approach has been to compare empirically observed networks with random graphs. The field of statistical graph theory where properties of random graphs are explored, is fundamentally linked to the Erdös-Rényi model of the late 1950s (Erdös and Rényi, 1959). The field was revolutionized in the late 1990s by the publication of two further models of random graphs: a model of small-world graphs (Watts and Strogatz, 1998) (uniting high local clustering with short average distances between nodes) and a model of random graphs with a broad (power-law shaped) degree distribution (Barabasi and Albert, 1999).

#### Defining the FU

Within Computational Neuroscience, the fundamental unit is typically defined as being individual neurons (Vladimirov et al., 2012) or on the level of cortical areas (Honey et al., 2009). The discussion below focusses on the latter case. In contrast to the discussion in (section Neuroscience) the fundamental unit is not necessarily identified with the cortical areas, but is more flexible, allowing aggregates of cortical areas or even abstract ones derived from the raw data (from fMRI) to be the fundamental units that constitute the nodes of a network. Such cortical areas can also be defined by anatomical means and neurobiological knowledge (as for example in the cortical areas of the cortical areas network of the cat or the macaque; see Hilgetag et al. (2000)) or as parcellations in terms of ‘regions of interest’ (as for example for the human connectome, see Hagmann et al. (2008)).

#### Separating SC and FC

In Computational Neuroscience, SC refers to brain network connectivity derived from anatomical (and other) data, at the level of the fundamental unit. The large-scale architectural properties (the ‘topology’) of a network resulting from SC may determine important dynamical and functional features of the system. FC refers to relationships among nodes inferred from the dynamics. Typical observables for FC are co-activations or sequential activations of nodes.

A striking example of network topology (SC) shaping dynamics (FC) is the following model (discussed in Müller-Linow et al. (2006)) which considers excitations propagating on a graph. A node can be excited (active), refractory (resting) and susceptible, waiting for an excitation in the neighbourhood. Upon the presence of such a neighbouring excitation, a susceptible node changes to the active state for a single time step, then goes into the refractory state, from which it moves to the susceptible state with a probability *p* at each time step. Furthermore, spontaneous excitations are possible with a small probability *f*. Running these dynamics on a graph with hubs and sparsely connected nodes (like the graphs from Barabasi and Albert (1999)), self-organised waves of excitations can be identified around the hubs (Müller-Linow et al., 2008). A broader perspective on such global, collective patterns formed by dynamics on graphs is provided in Hütt et al. (2014).

Within Computational Neuroscience, an important rather novel trend is to compare directly and quantitatively the relationship between SC and FC (SC/FC correlations). Here, correlations of structural brain connectivity with FC derived from the BOLD signal in fMRI studies show high correlation values in the range of 0.6–0.8 in simulations and around 0.3 in experimental data ( Honey et al. (2009); Garcia et al. (2012); Goni et al. (2014); Messé et al. (2015)). Such SC/FC correlations compare structural and FC only in one direction: network structure serves as a ’template’ for the self-organization of dynamical processes, resulting in a set of patterns characterised as FC. A more global perspective includes learning, i.e. the change of SC under the persistent action of FC. In its simplest form, such a co-evolution of structural and FC is given by Hebbian learning rules (Hebb, 1949), where – qualitatively speaking – frequently used network links persist, while rarely used links are degraded.

#### Understanding emergent behaviour

The co-evolution of SC and FC offers an interesting possibility for the overarching perspective of self-organization and emergent behaviours, as the system now can, in principle, tune itself towards phase transition points, maximizing its flexibility and its pattern formation capacities. This concept is called self-organised criticality and goes back to the pioneering work by Bak et al. (1987) (e.g., Chialvo, 2010; Moretti and Munoz, 2013; Hilgetag and Hütt, 2014). Phase transition points of a dynamical system are choices of parameters, which position the system precisely at the boundary between two dynamical regimes (e.g., steady states and oscillations). At such points a small change of the parameter value can induce drastic changes in system behaviour.

As already suggested with the example of waves around hubs, the concepts of self- organization and pattern formation may provide a useful theoretical framework for describing the interplay of SC and FC. Self-organization means that a set of elements under the influence of local inter-actions (described by ’control parameters’) creates long-range and frequently very complex structures (’patterns’), which cannot be described by the degrees of freedom of the individual elements but need to be assessed on the scale of the entire system (see, Hütt (2006) for more details and further references). The fact that such patterns emerge spontaneously, when critical values of the control parameters are passed, deeply links the concept of self-organization to phase transitions in dynamical systems Distinguishing between contributions to FC, which can be understood (and compared to SC) on the level of individual links, and contributions to FC, which are collective, global patterns on the graph (Hütt et al. 2014; see also the strategy from Morone et al., 2017), is a current challenge in understanding emergent behaviour.

#### Measuring connectivity

Returning to network topology, a wide range of descriptors of connectivity is used in Computational Neuroscience (and other disciplines). The term ’connectivity’ typically refers to the density of links in a graph (i.e., the number of links divided by the number of all possible links). Another common quantifier of connectivity is the average degree (i.e., the number of links divided by the number of nodes). Beyond these simple quantifiers, the connection pattern in a graph can be characterized in a multitude of ways, for instance via clustering coefficients (Watts and Strogatz, 1998), centrality measures (Newman, 2005) and the matching index or topological overlap (Ravasz et al., 2002; Goni et al., 2014), which evaluates the number of common neighbours of each pair of nodes. More global characterizations refer to the graph’s modular organization (Newman, 2006), possible hierarchies (Ravasz et al., 2002) and its composition of small sub-graphs (network ’motifs’; Alon, 2007).

### Geomorphology

Geomorphologists study the origin and evolution of landforms. Geomorphic surface processes comprise the action of different geomorphic agents or transporting media, such as water, wind and ice which move sediment from one part of the landscape to another thereby changing the shape of the Earth. Therefore, looking at potential sediment pathways (connections) and transport processes has always been one of the core tasks in Geomorphology. “Connectivity thinking” and related concepts have a long history in Geomorphology (e.g. Chorley and Kennedy, 1971; Brunsden and Thornes, 1979). However, since the beginning of the 21^st^ century connectivity research experienced a huge boom as geomorphologists started to develop new concepts on connectivity to understand better the complexity of geomorphic systems and system response to change. It is widely recognised that investigating connectivity in geomorphic systems provides an important opportunity to improve our understanding of how physical linkages govern geomorphic processes (Van Oost et al., 2000; Wainwright et al., 2011). Connectivity further reflects the feedbacks and interactions within the different system components under changing conditions (Beuselinck et al., 2000) and determines the propagation of the effects of change as flow pathways are modified and the structure of the landscape is transformed (Harvey, 2007; Lexartza-Artza and Wainwright, 2011). However, to date most - if not all - of the existing connectivity concepts in geomorphology represent a palimpsest of traditional system thinking based on general systems theory (e.g. von Bertalanffy 1968) rather than applying complex systems theory and related approaches that potentially provide the appropriate toolbox to study geomorphic system complexity.

#### Defining the FU

Landforms are the product of a myriad of processes operating at different spatial and temporal scales: defining a fundamental unit for the study of connectivity is therefore particularly difficult. Geomorphologists have traditionally drawn structural boundaries between the units of study which are often obvious by visible sharp gradients in the landscape, for example channel-hillslope or field boundaries. This imposition of structural boundaries has led to the separate consideration of these landscape compartments, rather than looking at the interlinkages between them, which results in an incomplete picture when it comes to explain large-scale geomorphic landscape evolution. Bracken et al. (2015) proposed a framework for understanding sediment transfer at multiple scales considering sediment connectivity as being “[…] the integrated transfer of sediment across all possible sources to all potential sinks over the continuum of detachment, transport and deposition […]” (Bracken et al., 2015; p. 179). However, this framework provides no insight into how the fundamental unit may be defined. Its size and demarcation is highly dependent on (i) the processes involved and (ii) the spatial and temporal scale of study (i.e. the timescale that constitutes a relevant event (cf. Bracken et al., 2015)). If, for example, the temporal scale of analysis is considerably greater than the frequency of key processes (i.e. a timescale that is sufficiently long to encompass sediment cascades in which all components of a catchment will be connected) then sediment connectivity will be perceived to be exceptionally high. Alternatively, if the temporal scale over which sediment connectivity is evaluated is less than the frequency at which key sediment-transport-related processes within the study domain operate, then sediment connectivity will be perceived to be lower (Bracken et al., 2015). The size of a fundamental unit in Geomorphology is thus dependent on the underlying research question and may range from plot- (e.g. single erosion rills) to mega-scale (e.g. landscape belts) (Slaymaker et al., 2009). However, geomorphic processes tend to vary between spatial scales, which leads to one of the key problems in geomorphology, i.e. scaling up processes measured at small spatial and temporal scales to explain large-scale geomorphic patterns and processes (cf. Bracken et al., 2015), for example, how to understand catchment-scale evolution from plot-scale erosion measurements.

Consideration of how fundamental units make up landscapes has a long history in geomorphology. Wooldridge (1932), for example, characterised topography as comprising facets of flats and slopes, while a richer characterization of a landscape fundamental unit is the “land element”, variously defined but always incorporating the notion of an area where the climate, parent material, topography, soil and vegetation are uniform within the limits significant for a particular application (for a more comprehensive overview see Mabbutt (1968) or Poeppl and Parsons (2017)). Most recently, based on the “land element” concept and by further integrating analogies from cell biology, Poeppl and Parsons (2017) defined so-called geomorphic cells as the fundamental unit of study in geomorphic connectivity studies. They conceptualise a geomorphic cell as being a three-dimensional body of the geomorphosphere, which is delimited from neighbouring cells and neighbouring spheres by different types of boundary, and types of connection called “connecteins”. Vertically, the upper boundary of a geomorphic cell is defined by the atmosphere, while the lower boundary is generally formed by the bedrock layer of the lithosphere. Laterally, geomorphic cells are delimited from neighbouring cells with a change in environmental characteristics that determine hydro-geomorphic boundary conditions (e.g. geology, soils, topography and/or vegetation).

#### Separating SC and FC

**Fig. 3:**
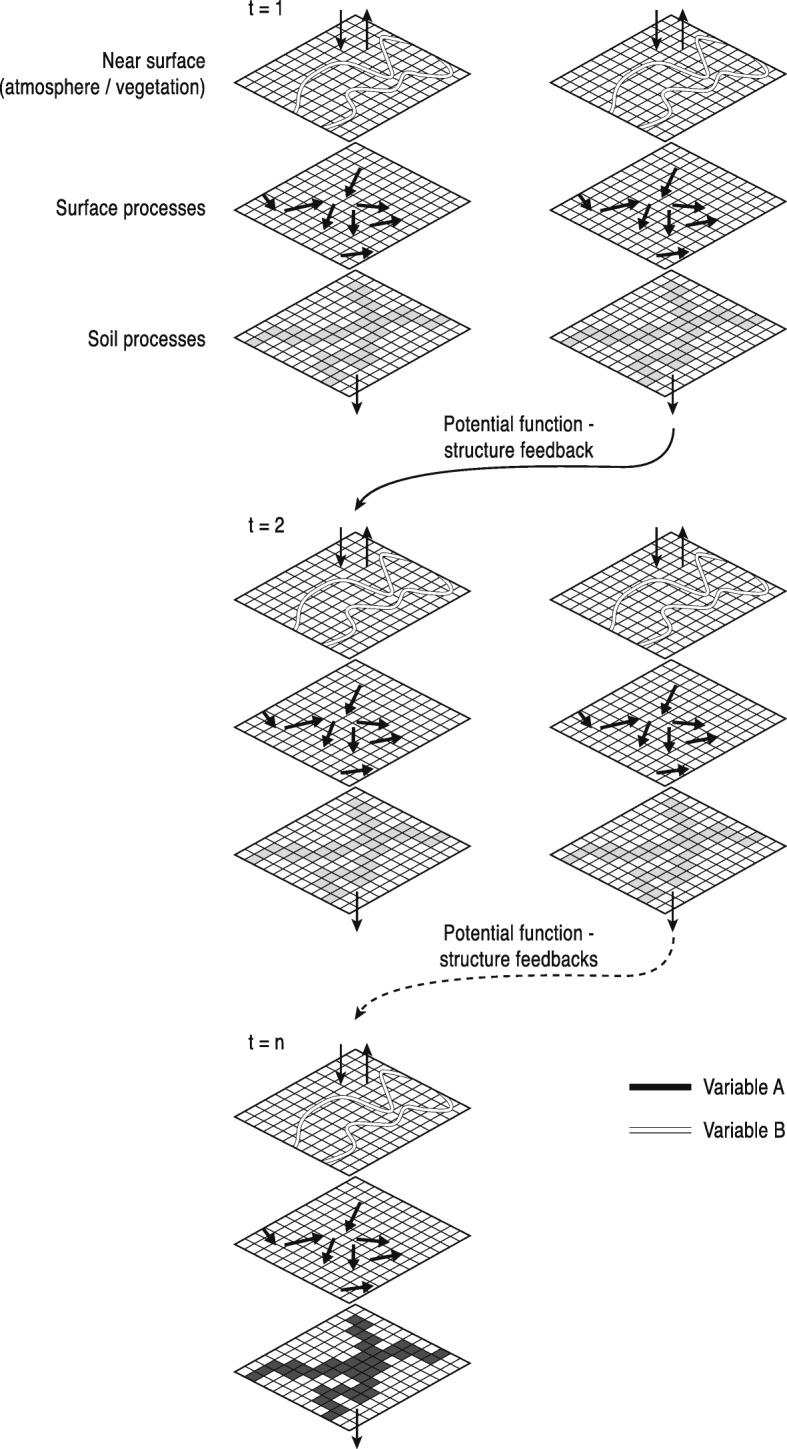
Geomorphic feedbacks between structural and functional connectivity. Schematic diagram of feedbacks between structural and functional connectivity (source: Wainwright et al., 2011). The relative locations of values of different variables which control SC may initially (t = 1) be quite discrete, leading to functional disconnections which are only connected during events of specific types or magnitudes, which in turn can create structural feedbacks by reorganizing landscape elements (e.g. vegetation, soil types). Through time (t = 2, …, n), these feedbacks may be reinforced so that structural and functional connectivity follow similar patterns, and the system become difficult to reverse (see Turnbull et al., 2008)

In Geomorphology, SC describes the extent to which landscape units (however defined) are physically linked to one another (With et al., 1997; Tischendorf and Fahrig, 2000a, b; Turnbull et al., 2008; Wainwright et al., 2011) whereas FC accounts for the way in which interactions between multiple structural characteristics affect the flow of material (Turnbull et al., 2008; Wainwright et al., 2011; Bracken et al. 2015). An early consideration of functional interlinkages between system compartments (i.e. hillslopes and river channels) was introduced by Brunsden and Thornes (1979) within their “landscape sensitivity” concept. However, besides a general notion of the importance of coupling between system components for landscape evolution the authors did not provide any further information on how to define and quantify these relationships. In Geomorphology it is becoming increasingly accepted that SC and FC cannot be separated from each other in a meaningful way due to inherent feedbacks between them (Fig. 3) (Turnbull et al., 2008; Wainwright et al., 2011; Bracken et al. 2015), and a conceptualization focussing on the linkages between process and (land)form has profound implications for the philosophy and methodology in Geomorphology.

Landscapes can be perceived as systems exhibiting a distinct type of memory, i.e. the imprint that geomorphic processes leave on structure which in turn governs future landscape processes, further acting on different spatial and temporal scales. Thus, a critical issue when separating SC and FC is determining the timescale at which a change in SC becomes dynamic (i.e., functional). Past geologic, anthropogenic and climatic controls upon sediment availability, for example, influence contemporary process-form relationships in many environments (Brierley, 2010) such as embayments (e.g. Hine et al., 1988; Phillips and Slattery, 2008) or human-impacted fluvial systems (e.g. Poeppl et al., 2017) on different spatial and temporal scales. In most geomorphic systems the imprint of memory and the timescales over which feedbacks affect connectivity are too strong for a separation of SC and FC. However, this philosophical position has not yet made its way into approaches to measuring connectivity. A challenge when developing quantitative descriptions of the structural-functional evolution of connectivity in geomorphic systems is thus how to incorporate memory effects. Furthermore, when distinguishing between SC and FC, the challenge is to achieve the balance between scientific gains and losses, further depending on the spatio-temporal scale of interest and the applied methodology.

#### Understanding emergent behaviour

The conceptualization of landforms as the outcome of the interactions of structure, function and memory implies that landscapes are organised in a hierarchical manner as they are seen as complex macroscopic features that emerge from myriad (microscopic) factors (processes) which form them at different spatio-temporal scales (Harrison, 2001). For example, river meander development (e.g. Church, 1996) or dune formation (e.g. Baas, 2002) can be seen as emergent properties of geomorphic systems that are governed by manifold microscale processes (e.g. microscale sediment transport events). In Geomorphology, emergence thus becomes the basis on which qualitative structures (landforms) arise from the (self-)organisation of quantitative phenomena (processes) (Harrison, 2001) operating at a range of different spatial and temporal scales. Due to conceptual and methodical constraints prediction and/or reconstruction of large-scale and long-term landscape evolution is difficult (i.e. the problem of up- and downscaling in geomorphology). In order to get a grasp on emergent behaviour of geomorphic systems recent advances in Geomorphology are based on chaos theory and quantitative tools of complex systems research (e.g. modelling approaches: e.g. Coco and Murray, 2007; combined approaches: e.g. D’Alpaos et al., 2007). Combining numerical models with new data-collection strategies and other techniques (as also discussed in 3.4.4), are assumed to represent a viable strategy to cope with the challenges as addressed above (cf. Murray et al., 2007). Nevertheless, the central question that arises is: “How far (if at all) are connectivity concepts and related methods useful when it comes to better understand emergent system behaviour?” It is hypothesized that multi-methodical approaches focussing on the interactions of SC and FC on different spatio-temporal scales might play a key role to understand better emergent behaviour of geomorphic systems. However, to date this hypothesis remains untested and is being subject to further inquiry. Yet, the potential appears to exist that connectivity may help to understand how geospatial processes produce a range of fluxes that come together to produce landscape form.

#### Measuring connectivity

In Geomorphology, it is only possible to measure (i) the morphology of the landscape itself from which SC is quantified or (ii) fluxes of material that are a result of FC and event magnitude. Few standard methods exist to quantify FC directly (Bracken et al. 2013). One of the key challenges to measure connectivity is to define the spatial and temporal scales over which connectivity should be assessed, which may depend on how the fundamental unit is defined. Another key challenge which also mainly arises from the geomorphic “scale problem” (see section Defining the FU) is the lack of standardized protocols for field-based quantitative appraisal of connectivity (Larsen et al. 2012; Okin et al. 2015) that would allow data comparison at multiple temporal and spatial scales. Furthermore, data comparability is often constrained by the measurement design (including the types of technical equipment involved).

Changes in SC can be quantified at high spatial and temporal resolutions using several novel methods that have been developed or improved over the past years. Structure-from-Motion (SfM) photogrammetry and laser scanning are techniques that create high-resolution, three-dimensional digital representations of the landscape. Sediment transport processes (FC) are traditionally measured using erosion plots for small-scale measurements to water sampling for suspended sediment and bedload traps in streams and rivers for large-scale measurements (e.g. Cerdà, 1997; García-Fayos, 1997; Wainwright et al., 2000; Boix-Fayos et al., 2006).

Recently, new techniques have been developed to trace and track sediment with higher spatial and temporal resolution. Sediment tracers, which can either occur naturally in the soil or be applied to the soil, have been increasingly used to quantify erosion and deposition of sediments. With these tracers, erosion and deposition can be determined at high spatial resolution at reasonably high accuracy (Guzmán et al., 2013; Parsons et al., 2014). Furthermore, laboratory experiments allow sediment tracking in high detail by using a combination of multiple high-speed cameras, trajectories and velocities of individual sand particles under varying conditions (Long et al., 2014). However, it is highly questionable if measuring water and sediment fluxes provides sufficient information to infer adequately FC, since these data solely represent snapshots of fluxes instead of reflecting system dynamics (incl. structure-function relationships) over long time scales.

Besides measuring landscape structure and sediment fluxes to infer connectivity, different types of indices and models are used. Connectivity indices mainly use a combination of topography and vegetation characteristics to determine connectivity (Borselli et al., 2008; Cavalli et al., 2013). These indices are static representations of SC, which are useful for determining areas of high and low SC within the study areas. Because indices are static, they do not provide information about fluxes. Different types of models (e.g. e.g. cellular automata: Baartman et al., 2013; Masselink et al., 2016a; process-based modelling: Mueller et al., 2007; statistical models: Poeppl et al., 2012; GIS approaches based on network theory: Heckmann and Schwanghart, 2013; Masselink et al., 2016b), on the other hand, do provide information on (potential) fluxes and can be powerful tools in determining how SC relates to sediment transport.

### Ecology

Landscapes are composed of interconnected ecosystems that mediate ecological processes and functions – such as material fluxes and food web dynamics, and control species composition, diversity and evolution. The importance of connectivity within ecology has been recognised for decades (e.g. Mac Arthur and Wilson, 1967), with the term “connectivity” being used to refer to the interaction between landscape structure and ecological function (e.g. organisms movement, transfer and transformation pathways of energy and matter) (Merriam, 1984) and the degree to which the landscape facilitates or impedes movement, transfer and transformation processes among patches or ecosystems (Taylor et al., 1993). Connectivity is now recognised to be an important determinant of many ecological processes (Kadoya, 2009) including population movement (Hanski, 1999), changes in species diversity (Cadotte, 2006), metacommunity dynamics (Koelle and Vandermeer, 2005) and nutrient and organic matter cycling (Laudon et al. 2011). For example, in marine ecology, identifying and quantifying the scale of connectivity of larval dispersal among local populations (i.e. the rate, scale, and spatial structure of successful exchange) is a fundamental challenge, since this drives population replenishment and therefore has profound implications for population dynamics, diversity and evolution of marine organisms (Cowen et al., 2006). However, since its initial use, the term ‘connectivity’ has been inconsistently defined (Calabrese and Fagan, 2004), with metapopulation ecologists seeking a habitat patch-level definition of connectivity, while landscape ecologists view connectivity as being a landscape-scale property (Merriam 1984; Taylor et al. 1993; Tischendorf and Fahrig 2000a, b; Moilanen and Hanski 2001). Regardless of the scale at which connectivity is defined within Ecology, there is nonetheless consensus that connectivity affects most population, community, and ecosystem processes (Wiens 1997; Moilanen and Hanski 2001).

#### Defining the Fundamental Unit

In Ecology higher levels of organization incorporate and constrain behaviour at lower levels (O’Neill et al., 1989). Hierarchy theory provides a clear tool for dealing with spatial scale, and suggests that all scales are equally deserving of study (Cadenasso et al., 2006). It is therefore critical that the fundamental unit be defined clearly as well as relationships that cross scales (Ascher, 2001). The fundamental unit is typically defined as being the ecosystem – a complex of living organisms, their physical environment, and their interrelationships in a particular unit of space (Weathers et al. 2013). In this respect, an ecosystem can be a single gravel bar, a whole river section, or the entire catchment, or an ecosystem can be a plant, a vegetation patch, or a mosaic of patches, depending on the spatiotemporal context and the specific questions. Hence, the ecosystem concept offers a unique opportunity in bridging scales and systems (e.g. aquatic and terrestrial, above- and belowground systems). Notably, this definition of the fundamental unit is scale-free; therefore identifying the fundamental unit will emerge naturally out of the ecosystem(s) in question. Whilst an appropriate definition of the fundamental unit is critical in Ecology, this does not present a challenge, as the ecosystem provides a clear-cut definition that is applied ubiquitously.

#### Separating SC and FC

Ecology has long been concerned with structure–function relationships (Watt, 1947), and connectivity now tends to be viewed structurally and functionally (Goodwin, 2003), taking both structure and function into account (often referred to as landscape connectivity; Belisle, 2005). Structural connectivity refers to the architecture and composition of a system (Noss and Cooperrider, 1994) (e.g. the size, shape, location and spatial arrangement patches; Calabrese and Fagan, 2004) and the physical relationships among these patches (Kadoya, 2009). Measurements of SC are sometimes used to provide a backdrop against which complex behaviour can be measured (Cadenasso et al., 2006). Functional connectivity depends not only on the structure of the landscape, but on the behaviour of and interactions between particular species, or the transfer and transformation of matter, and the landscapes in which these species and processes occur (Schumaker 1996; Wiens 1997; Tischendorf and Fahrig 2000b; Moilanen and Hanski 2001). Moreover, it is concerned with the degree and direction of movement of organisms or flow of matter through the landscape (Kadoya, 2009), describing the linkages between different landscape elements (Calabrese and Fagan, 2004). In terms of animals, the FC of a depends on how an organism perceives and responds to landscape structure within a hierarchy of spatial scales (Belisle, 2005), which will depend on their state and their motivation which in turn will dictate their needs and how much they are willing to risk to fulfil those needs (Belisle, 2005). Thus, the FC of a landscape is likely to be context and species-dependent (e.g. Pither and Taylor, 1998).

Linking and separating SC and FC is challenging. For example terrestrial and aquatic ecosystems are structurally and functionally linked through coupled biogeochemical cycles, and also through mechanisms related to behaviour and/or complex life cycles, as shown by many insect or amphibian species with life-history stages in both aquatic and terrestrial habitats. Furthermore, riverine assemblages are governed by a combination of local (e.g. habitat conditions) and regional (e.g. dispersal) processes. There is empirical evidence that the position within the river network (i.e. stream size) drives the composition and diversity of riparian plants, aquatic invertebrates and fishes. For example, in looking at the interacting effects of habitat suitability (patch quality), dispersal ability of fishes, and migration barriers on the distribution of fish species within a river network, it has been found that whilst dispersal is most important in explaining species occurrence on short time scales, habitat suitability is fundamental over longer time-scales (Radinger and Wolter, 2015). Hence, ignoring network geometry and the role of spatial connectivity may lead to major failure in conservation and restoration planning. Time lags may also persist in SC-FC relations, and may be due to ecological memory, or “legacy effects”, a term used in Ecology to describe the impacts of a species on abiotic or biotic features of a system that persist for a long time after the species has ceased activity, and which have an effect on other species (Cuddington, 2011; Lindborg and Eriksson, 2004; Volker and Van Allen, 2017). These legacy effects may consist of information (e.g. species life-history traits) or material (e.g. seeds or nutrients) (Johnstone et al. 2016). These time lags in the functional response to changes in system structure can confound the ability to make meaningful separations between structure and function.

#### Understanding emergent behaviour

Emergent behaviour in Ecology is evident by the scale-free nature of ecosystems. Because ecosystems can be defined at any scale (usually spatial rather than temporal), interactions across different hierarchical levels lead to emergent behaviour at a different scale too. A striking example of such emergent behaviour is the existence of patterns in vegetation, for example Tiger Bush (MacFadyen 1950, Clos-Arceduc 1956). However, although attempts to explain this phenomenon using advection-diffusion models (e.g. Klausmeier 1999, Couteron and Lejeune 2001, Hille-RisLambers et al. 2001) have successfully produced reasonably realistic (if somewhat idealised) patterns, they are often at the expense of realistic vegetation dynamics. A more extensive critique of such approaches is given in Stewart et al. (2014) who argued that rather than using model structures inherently designed to produce patterns in order to explain the existence of patterns, a connectivity-based process understanding is likely to produce greater insight into the emergence of vegetation patterns. Based upon the argument that spatial patterns emerge in response to interactions between landscape structure and biophysical processes (e.g. Turnbull et al. 2008), Stewart et al. (2014) used local, detailed process information to drive a connectivity-based model for vegetation patterns in the American Southwest.

Evolutionary impacts of past processes, such as glaciations also shape emergent behaviour in Ecology, through separations and reconnection of larger areas (even continents). Increases in physical connectivity of landscape patches also facilitate the invasion of non-native species which in turn may trigger long-term evolutionary processes for both native and non-native species (e.g. Mooney and Cleland, 2001). The challenge in Ecology is to overcome the highlighted methodological constrains to studying emergent behavior and develop approaches that truly allow for explorations of emergent behavior.

#### Measuring connectivity

Measuring SC tends to be based on simple indices of patch (or ecosystem) connectivity. Patch proximity indices are widely used (e.g. Bender et al., 2003), and are often calculated using remotely sensed imagery or ground-based measurements. Other structural approaches to looking at ecological corridors include landscape genetics, telemetry, least-cost models, raster-, vector- and network-based models, among many other methods, which offer unique opportunities to quantify connectivity (see Cushmann et al. 2013). Most metacommunity and metaecosystem studies apply lattice-like grids as landscape approximations, where dispersal is random in direction, and distance varies with species. However, many natural systems, including river networks, mountain ranges or cave networks have a dentritic structure. These systems are not only hierarchically organised but topology and physical flow dictate distance and directionality of dispersal and movement (Altermatt 2013, references therein). Larsen et al. (2012) proposed a directional connectivity index, which is a graph-based, multi-scale metric generalizable to different SC and FC applications. In a graph-based approach, patches (or habitats or ecosystems) are considered as nodes, which link pathways between these nodes. Most work in Ecology has focused on unweighted, one-mode (monopartite) networks (Dormann and Strauss, 2014).

Measuring FC requires dealing with complex phenomena that are difficult to sample, experiment on and describe synthetically (Belisle, 2005). Approaches to measuring FC have the greatest data requirements, and include connectivity measures based on organism movement, such as dispersal success and immigration rate, with, for example, a high immigration rate indicating a high level of FC. In a study on seven Forest Atlantic bird species, the SC-FC relation was explored using a range of empirical survey techniques (Uezu et al., 2005). Quantitative analysis of landscape structure was carried out using a suite of SC measures. Functional connectivity measures were derived from bird surveys and playback techniques, carried out at snapshots in time and at discrete locations. Whilst these empirical measures allow insight to SC-FC relations, they nonetheless go hand-in-hand with a series of assumptions that allow the level of FC to be inferred. Similarly, data on dispersal distances (a proxy for FC) also tends to be relatively sparse. For example, they have been collected for a small number of marine species (Cowen et al., 2006), and typically only for those species that have short larval durations (hours to days) and short distance dispersal (e.g. Sammarco and Andrews, 1989; Shanks et al., 2003).

An ongoing challenge associated with empirically-based studies for assessing FC in Ecology is that they provide only a snapshot of dispersal or migration, representing only one possible movement scenario. It is generally accepted that it is impossible to measure empirically the full range of spatial and temporal variability in FC (Cowen et al., 2006). Modelling approaches are being used increasingly to overcome the limitations of empirically-based approaches to measuring FC. However, these modelling approaches are still limited by a paucity of available empirical data to verify the results of modelling experiments. The limitations of patch-based or landscape-based approaches to studying connectivity, and the prevalence of ecological research being carried out at increasingly larger scales has driven research in the direction of using network-based approaches (e.g. Urban and Keitt, 2001), often drawing on the concept of modularity from Social Network Science, Physics and Biology, and using network-based tools from Statistical Physics that account for weighted (non-binary), directed network data (e.g. Fletcher et al., 2013). Progress has been made in developing network-based tools for analyzing weighted monopartite networks (e.g. Clauset et al., 2008), and more recently, weighted and two-mode (bipartite) networks have been used to study connectivity between different species. In weighted networks, the links between two species may be quantified in terms of their functional connectivity; i.e. the number of interactions observed, or the strength of interactions between different species (but not within the same species) inferred from collected data (Newman, 2004b; Dormann and Strauss, 2014). For example, in pollinator-visitation networks, pollinators interact with flowers, but pollinators do not interact among themselves (Vazquez et al., 2009). A major challenge in using weighted bipartite networks in Ecology is that many of the analytical tools available require one-mode projections of weighted bipartite networks (e.g. Martin Gonzalez et al., 2012), or unweighted (binary) bipartite networks (e.g. Guillaume and Latapy, 2004), meaning that potentially useful information of ecological connectivity is lost. However, tools are being developed to analyze weighted bipartite networks (e.g. Dormann and Strauss, 2014). Multi-layer networks are increasingly being used in Ecology with the advantage over simpler networks that they allow for analysis of inter-habitat connectivity of species and processes spanning multiple spatial and temporal scales, contributing to the FC of ecosystems (Timoteo et al., 2018). Advances are being made in the analysis of multi-layer ecological networks, with the recent developments in the analysis of modular structure of ecological networks (i.e. the extent to which a network is organised into cohesive groups (modules) of species that interact more strongly with each other than with other species Pilosof et al., 2017). A recent study, for the first time looked at modular structure (seed dispersal modules; i.e. communities of tightly interacting plants and their dispersers) across different habitats to look at the strength of connectivity between habitats (Timoteo et al., 2018). A strength of using multi-layer networks in the analysis of ecological systems is that it allows differentiation of intra-layer and inter-layer connectivity within the multi-layer network (Pilosof et al., 2017). Whilst multi-layer networks are potentially a valuable tool for measuring connectivity in ecological systems, the application of such tools is often limited by the amount of system complexity that can be sampled and analyzed, potentially leading to an over-simplification of real ecological networks (Kivela et al., 2014; Pilosof et al., 2017).

### Social Network Science

Social network scientists study the social behaviour of society including the relationships among individuals and groups. There is a long history of social network theory which views social relationships in terms of individual actors (nodes) and relationships (links) which together constitute a network. This history dates back to the development of the sociogram describing the relations among people by Jacob Moreno (1934). Later work by Leavit (1950), White (2008), Freeman (1979), Everett (1982), Borgatti (2005), and Wasserman and Faust (1994) created a foundation of social theory frameworks based on network analysis. In many cases the theory that was developed in understanding social systems was subsequently applied in fields such as ecology. One famous case is the ‘small world’ phenomenon noted by Stanley Milgram (1967) which later was used to describe information transfer in insects (Watts and Strogatz 1998). Social scientists have continued to lead the development in key areas with the statistical analysis of motifs (small building blocks found in networks) (Robins *et al.* 2007) and the evaluation of networks within a philosophical framework such as structuralism (Assiter 1984). In recent times the incorporation of ecological and social theory to facilitate socio-ecological analysis has expanded the social networks to include ecological systems (Janssen *et al.* 2006; Ekstrom and Young 2009). The focus on sustainability and resilience within these multifaceted networks continues to spawn novel solutions and advanced techniques (Bodin and Tengo, 2012; Kininmonth et al. 2015).

#### Defining the Fundamental Unit

Given that social network theory is often centred on the micro interaction of people there can be a convincing argument that the fundamental unit is the person (Wasserman and Faust 1994). Certainly many published networks in sociology are based on the interaction history of people within a small group (Sampson 1968; Zachary 1977). However with the advent of technology such as mobile phones, the internet, online gaming and social web pages (i.e. Facebook) this definition of the fundamental unit is less certain and some researchers now use the interaction itself as the unit of study (Garton *et al.* 1999). Ideas and behaviours that spread through a society (known as memes (Dawkins 1986)) or the use of textual analysis (Treml *et al.* 2015) have created networks that are abstracted from the individual. From a network perspective the individual human is not represented by a single node in these cases but instead might have temporary links to the ideas and behaviours that are in circulation. For example we are aware of the spread of technology, such as pottery styles across continents, but we remain unaware of the individuals involved. For many researchers the meso-scale focus on populations facilitates the analysis of organisational structures and their interactions (Ostrom 2009). This hierarchical nature of social interactions has resulted in an increased emphasis on organisational culture as a defining influence on the social network (Sayles and Baggio 2017a). Utilizing multi-layer networks to explore complex social theory promotes the conceptual possibility of combining fundamental units (Bodin 2017). For example the management of natural resources across a region requires a functioning social network within the management agencies (Bodin and Crona 2009; Kininmonth *et al.* 2015). However analysis of multi-layer networks that combine the fundamental units of organisation (often with cultural attributes) and individuals has demanded new methodological advances particularly in the interpretation of decision-making and engagement between the actors embedded within the associated organisation (Sayles and Baggio 2017a). In this regard the analysis of the diverse suite of roles that actors and organisations portray is highly topical in understanding the long- and short-term dynamics of social systems.

#### Separating SC and FC

**Fig. 4:**
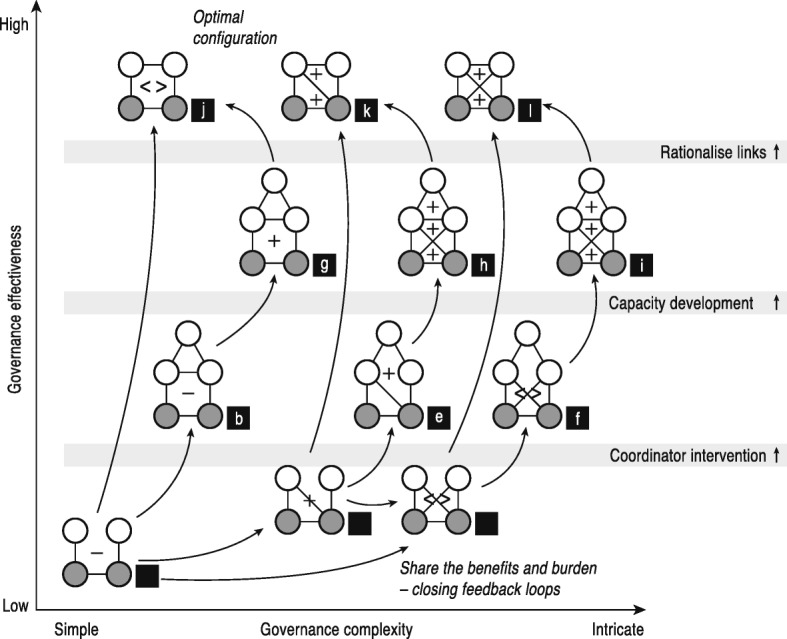
The relationship within the common resource pool motif subset display across effective-complexity space. This shows the various combinations of social interactions (white) that govern connected natural resources such as wetlands (grey). From Kininmonth et al. (2015)

The development of social networks has primarily been based on observed interactions between members of a group and these interactions have been used to generate structural networks. These networks have then been used to determine the basis for subsequent events, such as a split in the group, based solely on the distribution of links (Sampson 1968; Zachary 1977). For simple networks and simple events this approach appears to have merit, but when the networks become complex or highly dynamic this method is limited in terms of analytical power. To bridge the link to a more functional approach requires understanding the processes happening at the individual level such that the links have meaning at a functional level. One solution here is to understand the functional meaning of simple network structures (i.e. a triangle of 3 nodes and 3 links representing friends of friends; commonly referred to as motifs) found in the network. The powerful component is to try to recreate the larger network from the described frequency of specified motifs (Robins *et al.* 2007; Wang *et al.* 2013). This approach has significant statistical power rather than just qualitative comparisons and can be useful for many research objectives (Fig. 4). An alternative approach is to conduct experiments that seek to evaluate the individual’s response to a situation given variations in interaction structures (Baird *et al.* 2015). The difficulty with this method is translating the human response in an experimental setting, rather than real life, where the consequences are often of high impact. Otherwise the use of very large data sets, such as the phone calling patterns of millions of people, are providing insights into the dynamics of the network structures that function to respond to a given event (Barabási 2005). A more abstract approach is the use of cellular automata to describe the rules of local engagement and then observe the network responses in an artificial modelling environment (Wijermans and Schlüter 2014). Phenomena such as Small World topology has highlighted the widespread effect of structure and function on the larger network dynamics (Travers and Milgram 1969). Link prediction is also becoming widely used in social network studies to predict future interactions and the evolution of a network from the network topology alone (e.g. Liben-Nowell and Kleinberg, 2007).

Complicating the conceptual link between structure and function for social networks is the influence of culture. In particular, cultural norms are a strong influence on the responsiveness of social network structures such that different cultures are likely to generate different responses to identical network structures (Malone 2009; Stephanson and Mascia 2014). Key to this influence is the human propensity for diverse communication methods that have inflated the effect of memory on the function of interaction networks. This memory effect is also likely to affect the individual response following a repeat of the social interactions. Members of society will respond and interpret particular interactions differently based on their age group and background and this is evident in the expansion in computer-assisted social networks often binding diverse community groups (Garton *et al.* 1999). The complexity that an evolving mix of cultures brings to the analysis of social networks is a significant challenge to providing a general set of rules of social engagement across the planet.

#### Understanding emergent behaviour

**Fig. 5:**
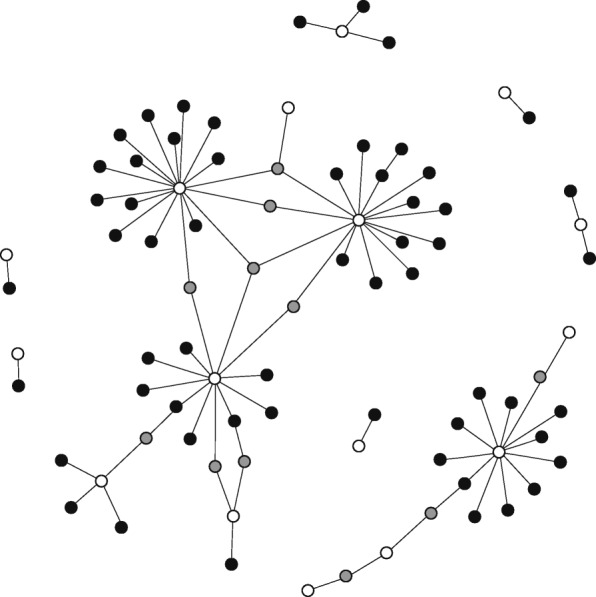
Network diagram of the interaction of fishers with people who buy fish. Black = single trade relationship, grey = multiple traders and white = traders. This network diagram highlights the emerging property of organised fishing businesses that are dependent on the access to capital. From Kininmonth et al. (2017)

The emergent behaviours observed within social networks has spawned many significant publications from the splitting of monks at an abbey (Sampson 1968) to the smoking habits of the general population derived from friendship clusters (Bewley *et al.* 1974; Christakis and Fowler 2008). The structure is observed and then the application of networking analysis tools, such as clustering, is used to determine the functional response (Barabási *et al.* 2002; Barabási 2005; Palla *et al.* 2007) (Fig. 5). The resilience of social systems is now seen as a direct response to the topological structure such as small world or scale free (Holling 2001). The translation of the resilience concept from a structural perspective involves maintaining the integrity of the network, despite this being difficult to predict or measure. Methods that impose a process on the nodes and links such as Susceptibility-Infection-Resistance for disease propagation can be highly dependent on density and centrality measures. The emergence of the network property such as resilience or effectiveness is conditional on the entire network interactions. To complicate matters further, the challenge of adopting models of social behaviour that recognise the diversity of social interactions across a population remains elusive.

#### Measuring connectivity

From the early research efforts of Moreno (1934) came the visual analysis of social networks using the depiction of people and interactions as nodes joined by links. Gradually the application of mathematics defined the various patterns observed. In particular, the work by Harary (1953) set up the foundation of structural analysis of social networks. The advent of fast computing was necessary to enable more dynamic analysis including the evaluation of networks against non-random networks. Centrality and link density measures formed the basis of many actor-level analytical tools (Garton *et al.* 1999) including cluster scrutiny but more sophisticated statistical approaches such as latent space (Hoff *et al.* 2002) were also applied in attempts to extricate the structure and function. The topological configurations that influence network function were incorporated into the analytical framework. Small world configurations, scale free systems and planar networks were found in many social systems (Barabási 2005, 2009). Motif analysis (Robins *et al.* 2007), especially the multivariate distribution of motifs, established their role through the use of Exponential Random Graph Models. This technique is still restricted in the configurations able to be utilised for analysis. The greatest challenge in the field of social network analysis is the extension of the analytical techniques to encompass the postulations of the socioecological paradigm. Understanding the links between natural resources and the corresponding social governance structures is critical for humanity’s management of depleted resources yet models remain simplistic and limited (Bodin *et al.* 2014). Understanding the heterogeneous networks across hierarchical systems within dynamic structures remains a subject of rapid development (Leenhardt *et al.* 2015).

Measuring connectivity in the social sciences is difficult due to ethical, practical and philosophical issues. These influences are found when collecting the data that describes the connectivity (Garton *et al.* 1999). Questionnaires that seek to record a range of social interactions are hampered by privacy (i.e. the identification of individuals), which is a frustration since individuals are pivotal to interpreting an observed change. Ethical considerations mean the use of publically collected data must remain anonymous and limited to the case in question. Tricking individuals to react through the use of physiological experiments can be fraught with danger as Stanley Milgram demonstrated. Another complication is the practical issue of who (and the organisation they represent) can conduct the interviews since people will respond differently to the type of person asking the questions based on their past interaction history or interview context (Garton *et al.* 1999). The alternative is collecting large data volumes on connecting behaviour such as mobile phones but this is limited to the numerical ID of the caller rather than a fully described demographic suite. In some cases the use of synthetic populations (Namazi-Rad *et al.* 2014), where the individual members of a theoretical society are constructed from a multifaceted demographic data, is one-way planners can circumvent the privacy issue. Philosophical considerations are required to understand the complex human responses to simple observations of connections. Applying a Marxist rather than a Durkheimian perspective will lead to different interpretations of the observed changes in social network structure (Calhoun 2002). However caching the network analysis in a particular school of thought is a powerful mechanism to reduce the vagueness of fundamental descriptions.

## Synthesis of key challenges

There are a number of important similarities in the way that the concept of connectivity is approached and the tools that are used within the disciplines explored. Notably though, there are also significant differences, which provides an opportunity for cross-fertilization of ideas to further the application of connectivity studies to improve understanding of complex systems. This section (i) evaluates the key challenges by drawing upon differences in the ways they are approached across the different disciplines (Table 1), enabling (ii) identification of opportunities for cross-fertilization of ideas and development of a unified approach in connectivity studies via the development of a common toolbox. We then (iii) outline potential future avenues for research in exploring SC-FC relations.

### Evaluating the key challenges

#### Defining the fundamental unit

Within all the disciplines explored the fundamental unit employed in any connectivity analysis depends on the spatio-temporal context of the study and the specific research question – this applies even where a clear fundamental unit might be self-evident (e.g., the individual in social network science, or the individual neuron or cortical area in neuroscience). The spatial and temporal scale of the fundamental unit may span orders of magnitude within a single discipline, and may thus have to be redefined for each particular study. For example, whilst for some applications in Neuroscience it is appropriate to adopt the neuron as the fundamental unit, for others the cortical area (many orders of magnitude larger in size) may be more appropriate – notably in cases where it becomes challenging to address adequately the connectivity of neurons due to computational limitations. This issue is also present in Geomorphology where adopting individual sediment particles as the fundamental unit would become too computationally demanding. In this sense, there are parallels between connectivity and the field of numerical taxonomy (Sneath and Sokal, 1973) where, despite the obvious taxonomic unit being the individual organism, an arbitrary taxonomic unit (termed an operational taxonomic unit) was employed. The exception to this general statement is the field of Ecology, where the ecosystem provides a conceptual unit that can be applied at any spatial scale. The concept of the ecosystem was introduced by Tansley (1935) and has been subject to much debate since. Despite the shortcomings of the ecosystem concept, within connectivity studies it is nonetheless useful to have an overarching concept that can be employed at any scale. The ecosystem concept is particularly useful when the interactions (connectivity) between different organizational levels are of interest, with an ecosystem at a lower hierarchical level forming a sub-unit of an ecosystem at a higher hierarchical level. Many systems are hierarchically organised, and therefore a key question for other disciplines is whether identifying something theoretically similar to the ecosystem concept may be useful. For many applications in connectivity studies, appropriate conceptualisation and operationalisation of the fundamental unit will depend on the purpose of investigations. For example where interventions within a system have the goal of managing or repairing a property of that system, the scale of the fundamental unit may be specified, to work within the certain system boundaries for a particular purpose. But as noted in the case of Ecology (section Defining the Fundamental Unit) it is critical that whilst defining the FU, relationships that cross scales are also defined clearly.

Although in most disciplines the fundamental unit corresponds to some physical entity, in Social Network Science for example, it may be more abstract, i.e. a unit of interaction. More abstract conceptualisations of the fundamental unit may be fruitful in other disciplines where the definition of a fundamental unit as a physical entity has proved difficult (e.g. Geomorphology), or in modelling approaches to examining connectivity (e.g. random graph models). Furthermore, the notion (in Systems Biology) that the fundamental unit is a concept dependent upon the current state of knowledge of the system under study is a valuable point that merits wider consideration.

#### Separating SC and FC

There is general consensus that SC is derived from network topology whilst FC is concerned with how processes operate over the network. In all the disciplines considered, the separation of SC from FC is commonplace, due to the ease with which they can be studied separately – especially in terms of measuring and quantifying connectivity. The success separating SC and FC in Systems Biology has been attributed to the fact that the structural properties and snapshots of biological function are typically measured in independent ways, whereas elsewhere it is common for FC to be inferred from measurements of SC. Whilst structural-functional feedbacks are widely recognised, the extent to which these feedbacks are explored/accounted for varies considerably, in accordance with factors such as how advanced the discipline is, and the sophistication of available tools within that discipline. Separating SC and FC, of course, imposes severe limitations on one’s ability to address these feedbacks. For example, in Geomorphology it is well established that structural-functional feedbacks drive system evolution and emergent behaviour, and whilst it is common in some applications to explore these feedbacks (e.g., landscape evolution models), there are still many applications where it remains common to look at relations between SC and FC in one direction only (i.e., the effect of structure on function). There is a similar tendency in Neuroscience to focus on structural-functional interactions rather than the full suite of reciprocal feedbacks between structure and function. However the increasing recognition within Geomorphology and Neuroscience of reciprocal feedbacks is heightening the need for additional tools that will allow the evolution of SC and FC and the development of emergent behaviour to be understood more fully. The importance of such feedbacks is highlighted in Computational Neuroscience, in the case where frequently used networks persist, whilst rarely used links are degraded leading to the development of network topology over time. Nevertheless, separating SC and FC does permit insights into the behaviour of systems insofar as it permits predictive models of function from structure that are amenable to experimental testing.

The ease and meaningfulness with which SC and FC can be separated will also depend on the timescale over which feedbacks occur within a system. Structural connectivity can only be usefully studied independently of FC if the timescale of the feedbacks is large compared to the timescale of the observation of SC. Any description of SC is merely a snapshot of the system. For that snapshot to be useful it needs to have a relatively long-term validity. Thus, for meaningful separations of SC and FC to be made, it is paramount to know how feedbacks work, the timescales over which they operate, and how connectivity helps us to understand these feedbacks. There are striking examples from several of the disciplines explored here of the ways in which feedbacks between SC and FC can lead to the co-evolution of systems towards a phase transition point – this is seen in Computational Neuroscience, and in Ecology and Geomorphology where system-intrinsic SC-FC feedbacks shift a system to an alternate stable state.

Linked to SC-FC relations and the validity of separating the two is the concept of memory. Memory is about the coexistence of fast and slow timescales. Qualitatively speaking, the length of distribution cycles in a graph can be viewed as (being related to) a distribution of time scales. Changes to SC in response to functional relationships imprint memory within a system. Thus, key questions are: How far back does the memory of a system go? Is memory cumulative? In systems subject to perturbations (possibly true for all discipline studied here) which perturbations control memory and its erasure? What are the timescales of learning in response to memory? In Neuroscience, the discipline which gives us the term ‘memory’, it is argued that the final imprint of memory is diffuse across the brain, and consequently difficult to assess. Other disciplines have similarly struggled to comprehend the instantaneous non-linear behaviour of their systems in terms of memory. In Ecology, ‘legacy effects’ make empirical approaches to the study of ecological interactions across space and time challenging (van der Putten *et al.*, 2009). In Social Network Science it is possible to speak of culture, which raises the notion of a hierarchy of memory effects on connectivity: one that has not yet been explored. In Geomorphology memory is related to feedbacks and/or thresholds – thus, exploring the coexistence of fast and slow timescales of processes and mechanisms is a potential avenue for future research. Of all the key challenges facing the use of connectivity, memory appears to be one which no discipline has yet resolved.

#### Understanding emergent behaviour

Emergence is a characteristic of complex systems, and is intimately tied to the relationship between SC and FC. The choice of fundamental unit will have implications for one’s ability to understand emergent behaviour, since connectivity at larger spatial scales emerges from connectivity at smaller spatial scales, and thus, microscopic units produce macroscopic behaviour through emergent properties. In this sense, a fundamental unit is an emergent property of microscopic descriptions.

An important question is how far does the analysis of connectivity help understand emergence? As noted in (section Understanding emergent behaviour), the co-evolution of SC and FC offers an interesting possibility for the overarching perspective of self-organization and emergent behaviours, as the system now can, in principle, tune itself towards phase transition points. Thus, by separating SC and FC in our analyses of connectivity, we remove the opportunity to understand and to quantify emergence – to understand how a system tunes itself towards phase transition points (and the role of external drivers). Without tools that can deal with SC and FC simultaneously, it is challenging to see how connectivity can be used to improve understanding of emergent behaviour. However, some suitable tools do exist. For example, adaptive networks that allow for a coevolution of dynamics on the network in addition to dynamical changes of the network (Gross and Blasius, 2008) provide a powerful tool that have potential to drive forward our understanding of how connectivity shapes the evolution of complex systems. Approaches are used in Computational Neuroscience that look at the propagation of excitation through a graph showing waves of self-organization around hubs, thus allowing exploration of conditions that lead to self-organised behaviour. However, even in this example, there is still great demand for new ideas that will more easily accommodate the study of memory effects (in all its various guises) and emergent properties. In Geomorphology and Ecology, key studies demonstrate how incorporating SC and FC into studies of system dynamics allows for the development of emergent behaviour (e.g. Stewart et al., 2014). However, such examples are relatively rare, which highlights the scope for trans-disciplinary learning which may help to drive forward our understanding of emergent behaviour.

Link prediction is a potentially useful tool that has been applied for example in Systems Biology and Social Network analysis. It can be used to test our understanding of how connectivity drives network structure and function (Wang et al. 2012; Zhang et al. 2013), and our understanding of emergent behaviour. If a comprehensive understanding of a system has been derived of the SC and FC of a network and their interactions, then we should be able to predict missing links (Lu et al., 2015). Lu et al. (2015) hypothesize that missing links are difficult to predict if their addition causes huge structural changes, and therefore the network is highly predictable if the removal or addition of a set of randomly selected links does not significantly change the networks structural features. Thus, prediction and network inference – even though blurring the distinction between SC and FC (see section Neuroscience) – can be used to identify the most important links in a network – i.e. where SC, FC and their interactions are most important.

#### Measuring connectivity

In view of the widespread adoption of the concept of connectivity it may seem surprising that actually measuring connectivity remains a key challenge. However, such is the case. Because connectivity is an abstract concept, operationalizing models into something measurable is not straightforward. The imperative here is to consider SC and FC separately. For the former, some disciplines (e.g. Geomorphology, Ecology) have developed indices of connectivity (e.g., Bender et al., 2003; Borselli et al., 2008; Cavalli et al., 2013). Such indices measure only SC, and their usefulness is a function of the timescales of the interaction of SC and FC. Furthermore, there is a concern as to what is the usefulness of such indices, other than as descriptions of SC: as might equally be said of clustering coefficients and centrality measures. To what extent can/do they enhance our understanding? Systems Biology, on the other hand, does not attempt to measure SC *per se*, but infers SC based on knowledge accumulation of the system. In that sense, connectivity may be seen as a means of describing current understanding. Neuroscience, in contrast again, measures connectivity directly through experimentation. How far such an approach could be applied in other disciplines raises the issue of ethics, as discussed in 3.6.4. Only in the case of Computational Neuroscience, which deals with analysed entities (the properties of which are defined *a priori*) is measuring SC straightforward.

Of the two, FC poses the greater measuring problem. In network terminology, FC may be thought of as the links in the network that are active, and is thus easier to derive if a network description of the system’s SC exists. Without such a description, FC can be derived from fluxes (e.g. movement of animals), but the measurement of fluxes may present its own difficulties (e.g., in Geomorphology).

Link prediction is also a potentially useful tool in deriving a network-based abstraction of a system where it is infeasible to collect data on SC and FC required to parameterise all links, or where links, by their very nature, are not detectable (Cannistraci et al., 2013). This problem of observability is inherent in Systems Biology where link types can be very diverse and it has already been noted that databases will drift in time. Therefore, the topological prediction of novel interactions in Systems Biology is particularly useful (Cannistraci et al., 2013). The use of link prediction also raises the possibility that data can be collected to represent the subset of a network (therefore reducing data collection requirements), and link prediction be used to estimate the rest of the network (Lu et al., 2015).

Separate, but directly linked to measuring connectivity, is analysis of the measurements. The most commonly applied approach is the use of graph theory. This powerful mathematical tool has yielded significant insights in fields as diverse as Social Network Science, Systems Biology, Neuroscience, Ecology, and Geomorphology. However, in many applications of network-based approaches simply knowing if a link is present or absent (i.e. a binary approach) is too basic or artificial, and characterising the capacity of a link or the relative significance of a link within a network is important. This issue can be dealt with by providing a more detailed representation of the network using weighted or directional links. The use of weighted links is common within network science (see for example Barratt et al., 2004), and is likely to be of particular importance when, for example, applying network-based approaches to hydrology where the capacity of flow pathway (channel) is an important structural element of links within the hydrological network (e.g. Masselink et al., 2016b). Using a weighted network can provide an additional layer of information to the characterisation of a network that carries with it advantages for specific applications, and to ignore such information is to throw out data that could potentially help us to understand these systems better (Newman, 2004a); hence, the importance of using measures that incorporate the weights of links (Opsahl and Panzarasa, 2009). More recently, further advances have been made in network-based abstractions of systems, for example, in Ecology, multi-layer networks are being increasingly used, which overcome the limitations of mono-layer networks, to allow the study of connections between different types (or layers) of networks, or interactions across different time steps. Similarly, bipartite networks have been used to provide a more detailed representation of different types of nodes in a network. These more complex network-based approaches carry with them advantages that a more detailed assessment of connectivity within and between different entities can be assessed. However, whilst there are many advantages in using more complex network-based abstractions of a system (weighted, bipartite and multi-layer networks), there are also inherent limitations as many of the standard tools of statistical network analysis applicable to binary networks are no longer available.

In the case of weighted networks, even the possibility of defining and categorizing a degree distribution on a weighted network is lost. In some cases there are ways to modify these tools for application to weighted networks, but one loses the comparability to the vast inventory of analysed natural and technical networks available. A further problem of assigning weights to network links is that it requires greatly increased parameterisation of network properties, which may in turn start to drive the outcome of using the network to help characterise SC and FC and may influence any emergence we might have otherwise seen. However, in recognition of not throwing away important information associated with the weights of links, there are increasingly tools available to deal with weighted links, including: the revised clustering coefficient (Opsahl and Panzarasa, 2009); node strength (the sum of weights attached to links belonging to a node) (Barratt et al., 2004); average node strength (the average strength, *s*, of nodes of degree *k*, i.e. *s = s(k)*, which describes how weights are distributed in the network) (Menichetti et al., 2014); and the inverse participation ratio (the average inverse participation ratio of the weights of the links incident upon nodes of degree *k, i.e. Y = Y(k)*, which describes how weights are distributed across the links incident upon nodes of degree *k*) (Menichetti et al., 2014).

As already discussed in the case of Ecology, a limitation of bipartite networks is that to analyze these networks, a one-mode (monopartite) projection of the network is required, as many of the tools available for monopartite networks are not so well developed for bipartite networks. An important issue when analyzing bipartite networks is therefore devising a way to obtain a projection of the layer of interest without generating a dense network whose topological structure is almost trivial (Saracco et al., 2017). Potential solutions to this issue include projecting a bipartite network into a weighted monopartite network (Neal, 2014) and only retaining links in the monopartite projection by only linking nodes belonging to the same layer that are significantly similar (Saracco et al., 2017). A further issue is that it is often not possible to recover the bipartite graph from which the classical form has been derived (Guillaume and Latapy, 2006). Developments are being made in our ability to analyze bipartite networks directly; for example, progress has been made in developing link-prediction algorithms applicable to bipartite networks (e.g. Cannistracti et al., 2013).

Similarly, to apply standard network techniques to multi-layer networks requires aggregating data from different layers of a multi-layer network to a mono-layer network (De Dominico et al., 2013) which can result in a lot of valuable information being discarded (Kivela et al., 2014), although approaches are being developed to reduce the amount of information loss (De Dominico et al., 2014).

Careful consideration of the most appropriate tools is thus required when measuring connectivity using a network-based abstraction. Key questions are: Is it practical/doable to collect the data required to parameterise the network-based model of the system? Can a sensible projection of a bipartite network be derived, to facilitate analysis of the network? Is it possible to derive a monoplex abstraction of a multiplex network without losing too much information?

### A unified approach in connectivity studies: Development of a ‘common toolbox’

From this review it clear that the persistence of the four key challenges identified depends on the availability of different types of tools and their varied applications across the disciplines (Table 1). Notably, disciplines that are more advanced in their application of network-based approaches appear to be less limited by the four key challenges. The conceptual similarities in SC and FC observed between the disciplines discussed here, in which a wide range of different types of systems can be represented as nodes and links (Fig. 1) presents the ubiquitous possibility for the more general study of SC and FC using network-based approaches, even in disciplines where such an approach is not commonplace.

**Fig. 6:**
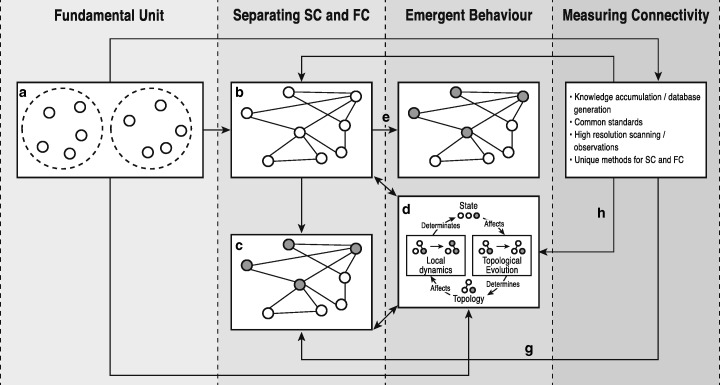
Network-centred common toolbox. Diagram showing how a network-centred common toolbox implicitly addresses the four (inextricably linked) key challenges: defining the fundamental unit, separating SC and FC, understanding emergent behaviour and measuring connectivity. A. Groups of nodes form fundamental units at higher levels of organization (denoted by grey dashed lines); B. Topological representation of system structure (spatially embedded depending in the system in question); C. Identifying parts of the network that are dynamic (functionally connected); D. Adaptive network where the evolution of topology depends on the dynamics of nodes (source: Gross and Blasius; 2008). Network adaptation at multiple (cross scale) levels of organization shapes emergent behaviour; E. FC may have an emergent aspect (self-organised, collective patterns on the structural network) that is independent of network adaptation; F. The fundamental unit should dictate the measurement approach; G. Measurements of SC and FC should be used to parameterise and test network-based representations; H. How we measure connectivity determines our ability to detect how connectivity leads to emergent behaviour

To pave the way forward in research into complex systems using connectivity approaches, we propose that a ‘common toolbox’ be compiled that can be applied across different disciplines to understand better the dynamics and characteristics of complex systems and the emergence of whole-system behaviour (Fig. 6). This common toolbox can be employed across the different disciplines to solve a set of common problems. Network-based approaches drawing upon the tools of Graph Theory and Network Science reside at the core of this common toolbox as they have been applied in disciplines where the key challenges pose less of a problem. In combination with network-based approaches, high resolution imaging/measurements of system dynamics (structural/functional developments), common standards for measuring connectivity, and continued knowledge accumulation, and the use of independent approaches to characterise SC and FC also reside at the centre of this common toolbox and assist with the development of suitable network-based representations of SC and FC.

A common toolbox requires that tools are readily accessible. The widespread uptake of the tools of Graph Theory has been facilitated by the implementation and dissemination of various graph theoretical models. Facilitating this uptake is the freely available stand-alone open source packages or enhanced parts of more general data analysis packages, all of which are becoming more sophisticated with time. A common toolbox can draw upon many existing freely available tools. One example is the Brain Connectivity Toolbox (Rubinov and Sporns, 2010) which was developed for complex-network analysis of structural and functional brain-connectivity data sets using the approaches of graph theory. More recently this toolbox has been used to investigate braided river networks (Marra et al., 2013), as well as channel networks within lava flows (Dietterich and Cashman, 2014). Similarly, other freely available connectivity metric tools from neuroscience have been used to assess structural and functional hydrologic connectivity, although further challenges have been noted, including issues concerning results interpretation including the occurrence of ‘false’ FC in the absence of SC, a strong dependence of results on chosen interpretation thresholds and the choice of appropriate length and sampling frequency of input data time series (related to scale issues) (see Rinderer et al. 2018).

Core foundations of the common toolbox upon which tools can be applied/abstractions can be made to facilitate suitable measurements are:

1. **Continued knowledge accumulation.** This enables the fundamental unit to be defined based on the system in question, which is then represented within the network as a node. To deal with multi-scale dynamics within a system, groups of nodes at one level of organization can form a fundamental unit at a higher level of organization.
2. **Network-based approaches.** These are well suited to the separation of SC and FC through the topological representation of system structure (SC) and through identifying parts of the network that are dynamic (FC). The spatial embeddedness of many networks is an essential feature, whereby the location of nodes and their spatial proximity is an important feature of the system, and it is necessary that this be accounted for. Further, the position of nodes within a network or node characteristics may alter the relative weighting of links.
3. **Accounting for network adaptation.** In recognition that SC-FC relations evolve (potentially leading to emergent behaviour), accounting for network adaption, where the evolution of network topology depends on node dynamics, is essential (Gross and Blasius, 2008). Only by dealing with network adaptation can SC-FC feedbacks and interactions be dealt with. Also important for understanding emergent behaviour is the capacity for fundamental units to be represented at multiple levels of organization, since this is critical where emergent behaviour is the result of cross-scale interactions and feedbacks.

Whilst connectivity research in complex systems should not be restricted to the use of a single tool or approach, there are clearly advances that can be made in connectivity studies by merging tools used within different disciplines into a common toolbox approach and learning from examples from different disciplines where certain challenges have already been overcome. It is important to recognise that not all the tools of the common toolbox will be applicable to all applications in all disciplines, and that some disciplines will only require a subset of approaches. Furthermore, it is important not to overcomplicate analyses, for instance through the use of spatially embedded networks where space is not an important network characteristic, or through the use of weighted links in cases where this is not critical to the representation of a system. Overcomplicating network representation reduces the scope for some network-based metrics to be used to quantify connectivity (e.g. you lose the capability of being able to define a degree distribution in a weighted network).

To operationalise this common toolbox, what is required now is a transdisciplinary endeavour that brings together leading scholars and practitioners to explore applications of connectivity-based tools across different fields with the goal of understanding and managing complex systems. Examples include: (i) determining how critical nodes shape the evolution of a system and how they can be manipulated or managed to alter system dynamics; (ii) deriving minimal models of SC and FC to capture their relations and identify the most relevant properties of dynamical processes, and (iii) to explore how shifts in network topology result in novel systems. Key to fulfilling this goal will be: synthesising theoretical knowledge about structure-function connectivity (SC-FC) relationships in networks; exploring the ranges of validity of SC-FC relationships and reformulating them for usage in the application projects; deriving suitable (minimal) abstractions of specific systems, such that the tools within the common methodology become applicable. Also important will be the synthesis of distinct methods that are similar in terms of the theoretical basis and share common ways of quantitatively describing specific aspects of connectivity. An important task will be to test the applicability, compatibility and enhancement of consistent methods in the common toolbox from one discipline to the other. Then, using the common toolbox, it will become possible to explore and understand commonalities in the structure and dynamics of a range of complex systems and hence of the respective concepts that have been developed across scientific disciplines.

### Future avenues for research in exploring SC-FC relations

In addition to these findings, other areas that may yield novel insights into SC-FC relations and assist in understanding commonalities in the structure and dynamics of a range of complex systems can be highlighted. That some of these specific areas have already been explored in some disciplines, but not all, presents an opportunity to investigate if they are evident across complex systems more generally, and provide opportunity to build upon the core foundations of the common toolbox outlined in (section A unified approach in connectivity studies: Development of a ‘common toolbox’). Examples include:

1. **Estimating the importance of certain network components using the elementary flux mode concept.** The importance of certain network components has been demonstrated in Systems Biology, but there are opportunities for all disciplines using network-based approaches to identify which parts of systems (networks) are particularly important. In Systems Biology elementary mode analysis is used to decompose complex metabolic networks into simpler units that perform a coherent function (Stelling et al., 2002), for example, the minimal set of enzymes that can support steady-state operation of cellular metabolism. Thus, there is opportunity to extend the concept of elementary mode analysis to other disciplines to predict key aspects of network functionality.
2. **Exploring short-range versus long-range connectivity in spatially embedded networks.** Short range versus long range connectivity has been highlighted as being important in many real networks, with the lengths of links characterised by a power-law distribution (Daquing et al., 2011). As seen in the case of Social Network Science, Social-Ecological Studies and Geomorphology, there are striking situations in which the proximity of structurally connected nodes influences the FC of the network. For example, geographic proximity facilitates the creation and maintenance of social networks (e.g. Preciado et al., 2012; Meyners et al., 2017) due to the reduced effort required to maintain social ties, although communication technologies and social networking platforms are changing the effect that geographic proximity has on social relations (e.g. Koban and Kruger 2018). Geomorphic landscape units tend to be more frequently connected by fluxes of materials the closer they are. In social-ecological systems, the underlying environmental system should not be divorced from its social context (i.e. networks of people and organizations), although often there is the issue of a spatial-scale mismatch between resource management structures and environmental systems (Sayles and Baggio, 2017b). Thus, the spatial dimension of networks plays a role in determining the functioning of the network and importantly, the systems’ behaviour especially near a critical point (i.e. threshold) (Daquing et al., 2011). Whilst the spatial characteristics of networks are widely recognised as being important, in some disciplines, they are still often not accounted for in network-based approaches. For example in the brain where cortical wiring is to some extent distance dependent (Kalisman et al., 2003), the spatial properties of the network are likely to be important since cortical networks have a spatial dimension (Voges et al., 2007). However, within Neuroscience many studies assume a random graph approach with completely random wiring (Brunel, 2000) which disregards the spatial dimension. Thus, in systems where the spatial characteristics of connectivity are important, accounting for short- and long-range connectivity and spatial characteristics of the network may be critical to understanding the behaviour of the system, and may yield important insights to understanding threshold dynamics. Selecting the appropriate network analysis tools when space is important is thus essential.
3. **Exploring power law relations of SC and FC.** In Systems Biology power law degree distributions of metabolic networks have been observed to match a power law degree distribution of metabolic fluxes. However other disciplines have not explored if law degree distributions in measures of SC correspond to power law degree distributions in FC; doing so may yield insights into SC-FC relations that might improve understanding of the evolution of network topology and system dynamics, and may help understand emergent behaviour.
4. **Identifying hump-shaped SC-FC relations:** There are examples in Ecology, Geomorphology and Systems Biology where SC-FC relations are hump-shaped. For example in Ecology connectivity-diversity relationships are often hump shaped, with floodplain biodiversity peaking in areas with an intermediate degree of hydrological connectivity (Ward et al., 1999). Identifying systems or networks that have a hump-shaped structure-function relationship could yield important insights into SC-FC feedbacks and threshold dynamics (i.e. reverse changes in FC once a structural threshold has been passed).

Although it is fairly common to borrow and adapt tools and concepts from one discipline to another, it is important to establish which methods are best suited to a particular discipline. It is important to consider which element or aspect of connectivity is transferable between disciplines – is it methods used to deal with SC and FC in connectivity analysis, or tools used to measure connectivity, or something more substantive in theoretical terms? For example, there may be issues when using concepts from ecology to explain human sub-systems, as humans are self-reflective and anticipatory. Furthermore, there may be issues, for example when transferring theories and concepts relating to patterns in Physics and Chemistry to patterns in Biology which have often undergone a clear evolutionary tuning and might serve a system level function, unlike patterns in Physics and Chemistry which are often just a by-product of nonlinear interactions of system components (see section Understanding emergent behaviour). Nevertheless, there is certainly great possibility to transfer tools and concepts between disciplines, as long as care is taken to ensure the suitability and applicability of such transfers.

## Summary

Across a range of disciplines connectivity has been a transformative concept in understanding and describing what are considered to be complex systems. Although conceptualisations and operationalisations of connectivity have evolved largely within their disciplinary boundaries, we have shown both that similarities in the concept of connectivity and its application among disciplines are also evident, and that common problems in using connectivity science are present.

In some disciplines there are standard practices for understanding, measuring, monitoring and modelling connectivity. It is clear, therefore, that progress can be made across all disciplines by learning from each other to advance the use of connectivity to understand our specific topics. This learning from each other can be facilitated through the use of the common toolbox, which will facilitated a greater degree of co-operation and cross-fertilization among these disciples, particularly in terms of developing common tools to analyse connectivity.

## Abbreviations

BOLD: Blood Oxygenated level Dependent
EEG: Electroencephalography
FC: Functional Connectivity
fMRI: Functional Magnetic Resonance Imaging
MEG: Magnetoencephalography
PET: Positron emission tomography
SC: Structural Connectivity

## Footnotes

1 Karl Zilles, private communication
